# Assessment of Excess Mortality and Household Income in Rural Bangladesh During the COVID-19 Pandemic in 2020

**DOI:** 10.1001/jamanetworkopen.2021.32777

**Published:** 2021-11-15

**Authors:** Prabhat Barnwal, Yuling Yao, Yiqian Wang, Nishat Akter Juy, Shabib Raihan, Mohammad Ashraful Haque, Alexander van Geen

**Affiliations:** 1 Department of Economics, Michigan State University, Lansing; 2 Department of Statistics, Columbia University, New York, New York; 3 Innovations for Poverty Action, Bangladesh, Dhaka, Bangladesh; 4 Lamont-Doherty Earth Observatory, Columbia University, Palisades, New York

## Abstract

**Importance:**

A slow or incomplete civil registry makes it impossible to determine excess mortality due to COVID-19 and difficult to inform policy.

**Objective:**

To quantify the association of the COVID-19 pandemic with excess mortality and household income in rural Bangladesh in 2020.

**Design, Setting, and Participants:**

This repeated survey study is based on an in-person census followed by 2 rounds of telephone calls. Data were collected from a sample of 135 villages within a densely populated 350-km^2^ rural area of Bangladesh. Household data were obtained first in person and subsequently over the telephone. For the analysis, mortality data were stratified by month, age, sex, and household education. Mortality rates were modeled by bayesian multilevel regression, and the strata were aggregated to the population by poststratification. Data analysis was performed from February to April 2021.

**Exposures:**

Date and cause of any changes in household composition, as well as changes in income and food availability.

**Main Outcomes and Measures:**

Mortality rates were compared for 2019 and 2020, both without adjustment and after adjustment for nonresponse and differences in demographic variables between surveys. Income and food availability reported for January, May, and November 2020 were also compared.

**Results:**

Enumerators collected data from an initial 16 054 households in January 2020; 14 551 households (91%) responded when contacted again by telephone in May 2020, and 11 933 households (74%)responded when reached again over the telephone in November 2020, for a total of 58 806 individuals (29 726 female participants [50.5%]; mean [SD] age, 26.4 [19.8] years). A total of 276 deaths were reported between February and the end of October 2020 for the subset of the population that could be contacted twice over the telephone, slightly below the 289 deaths reported for the same population over the same period in 2019. After adjustment for survey nonresponse and poststratification, 2020 mortality changed by −8% (95% CI, −21% to 7%) compared with an annualized mortality of 6.1 deaths per 1000 individuals in 2019. However, in May 2020, salaried primary income earners reported a 40% decrease in monthly income (from 17 485 to 10 835 Bangladeshi Taka), and self-employed earners reported a 60% decrease in monthly income (23 083 to 8521 Bangladeshi Taka), with only a small recovery observed by November 2020.

**Conclusions and Relevance:**

In this study of households in rural Bangladesh, all-cause mortality was lower in 2020 compared with 2019. Restrictions imposed by the government may have limited the scale of the COVID-19 pandemic in rural areas, although economic data suggest that these restrictions need to be accompanied by expanded welfare programs.

## Key Points

**Question:**

Is the low COVID-19–related mortality reported in Bangladesh for 2020 associated with massive undercounting?

**Findings:**

This repeated survey study including households from a sample of 135 villages in rural Bangladesh found that all-cause mortality in the surveyed are was lower in 2020 compared with 2019, but measures to control the COVID-19 pandemic were associated with a reduction in rural income and food availability.

**Meaning:**

These findings suggest that government restrictions designed to curb the spread of COVID-19 may have been effective in 2020 but needed to be accompanied by expanded welfare support.

## Teaser

This survey study examines the association of the COVID-19 pandemic with excess mortality and household income in rural Bangladesh in 2020.

## Introduction

Excess mortality is a measure of the net impact of COVID-19 that can be used to compare countries with different reporting systems and different definitions of COVID-19 deaths.^1,2^ A key requirement is that mortality data are reliably compiled within a reasonable time frame through a country’s civil registration system. This is not the case in most low-income countries, where underreporting of COVID-19 deaths in 2020 is, therefore, difficult to assess.^3,4,5^ The lack of reliable excess mortality data in low-income countries means that citizens as well as policy makers cannot gauge the trade-off between the economic costs and public health benefits of restrictions.^6,7^

During the year that ended March 1, 2021, a total of 8400 deaths in Bangladesh had been officially attributed to COVID-19.^8^ This corresponds to 1% of 820 000 annual deaths in the country in a normal year calculated from a population of 167 million and an annual mortality rate of 4.9 deaths per 1000 individuals.^9^ However, only one-third of deaths are currently officially recorded within 45 days in Bangladesh.^10^ The number of COVID-19 deaths could, therefore, have been considerably higher than national data suggest.^11,12^

**Figure 1.**
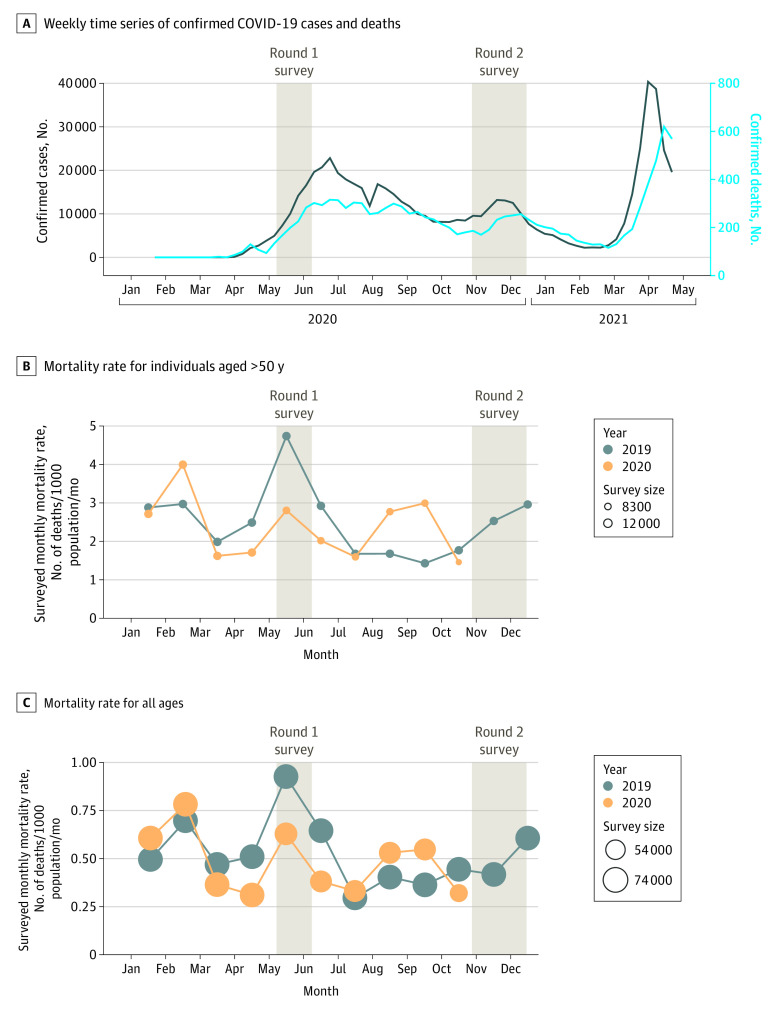
Variations in COVID-19 Cases and Deaths Over Time A, Weekly time series of confirmed COVID-19 cases (black line) and deaths (blue line) in Bangladesh in relation to timing of our telephone surveys, from February 2020 to April 28, 2021. B and C, Monthly sample mortality rate for elderly individuals (aged >50 years) and all ages in 2019 and 2020 in study villages. The mortality rate is calculated relative to the size of the survey sample for that month, which is indicated by the dot size. The times series of mortality rates ends in the last fully covered month of October 2020 preceding the second round of telephone calls. The highest mortality rate recorded in May 2019 could be an artifact of the first telephone survey being conducted in May 2020 and households reporting that a member died approximately 1 year ago.

According to government data, a majority of COVID-19 cases confirmed by polymerase chain reaction testing were concentrated in 2020 around the capital Dhaka and the next largest city Chattogram (eFigure 1 in the Supplement). Thousands of cases have also been reported from more remote districts, however. In spite of a de facto lockdown declared on March 23, 2020, the number of confirmed cases increased rapidly to exceed 20 000 per week in June 2020 (Figure 1A). Over much of the summer, the number of deaths attributed to COVID-19 was approximately 250 per week. Both cases and deaths then gradually decreased through February 2021, with the exception of a temporary resurgence in November 2020.

The goal of this study was to determine whether the relatively low number of officially reported COVID-19 deaths in rural Bangladesh in 2020 compared with other countries could have been associated with massive undercounting.^5,13,14^ Using detailed survey data containing individual-level information for a sizeable population, we estimated month-, age-, sex-, and education-specific mortality. We calculated excess mortality relative to 2019 after adjusting for key characteristics and averaging over the January 2019 census population. We complemented the analysis with data on the economic impacts associated with the pandemic obtained during the same telephone surveys.

## Methods

 This survey study was approved by the institutional review boards at both Michigan State University and Columbia University. The field research was implemented in Bangladesh by Innovations for Poverty Action, with ethics review provided by a reliance agreement with the Michigan State University institutional review board.

### Census Survey

Using an approach developed for conflict zones,^15^ we inferred mortality in our study area in 2020 by repeatedly conducting a census of a large number of households, once in person in early 2020 and twice over the telephone later in the year. The original purpose of the study was to compare the effectiveness of various ways of delivering the outcome of well tests for arsenic to reduce exposure from drinking contaminated well water (eFigure 1 in the Supplement). We explained the objective of the arsenic study to each respondent during the in-person census survey and recorded their verbal consent. During the telephone surveys, the modification to evaluate the impact of COVID-19 was explained in general terms and informed consent was recorded again.

Repeated census surveys were conducted in 135 villages, or predefined portions (*paras*) of larger villages, in a densely populated 350-km^2^ rural area of Bangladesh located 30 to 100 km to the northeast of Dhaka (eFigure 1 in the Supplement). Proxies for socioeconomic status, such as education and the number of rooms in the house,^16^ were no different in the study villages compared with other rural areas of the country (eTable 1 in the Supplement). The proportion of the population engaged in agriculture in the study villages was somewhat lower, however.

From January 15 to February 3, 2020, enumerators went door-to-door to contact all 17 538 households identified in the study villages or *paras*. Sharing a kitchen was the criterion used to define a household. Among these households, 1478 were absent and could not be reached (eFigure 2 in the Supplement). Of the remaining 16 060 households, only 6 declined to participate in the original study. After consent was obtained, the name, age, sex, and relationship of each individual member of the household, as well as GPS coordinates of each house and up to 2 mobile telephone numbers, were recorded electronically (eAppendix in the Supplement).

During the first telephone survey conducted May 8 to June 7, 2020, a total of 14 551 households (91%) surveyed in person in January 2020 could be contacted and consented to respond. During the second telephone survey conducted October 27 to December 14, 2020, 11 933 households (74%) consented. A total of 11 256 households (70%) were reached during both telephone surveys. Single-member households, 342 in all, require special consideration because a death during the study period could not have been reported by that person. The total population of consenting households surveyed in person in January 2020 amounts to 81 164 individuals. This number is used as the reference population and includes 7921 household members recorded during the telephone surveys who had reportedly been overlooked in January 2020, as well as 484 deaths in the households that occurred reportedly in 2019. The reference population does not include 1068 household members who joined the household from elsewhere in 2020. This study follows American Association for Public Opinion Research (AAPOR) reporting guideline for survey studies (eTable 2 in the Supplement).^17^

Respondents were asked over the telephone whether a reported death was the result of injury (eg, road collision injuries) and whether treatment from a doctor or at a hospital was sought. Respondents were also asked whether death was preceded by symptoms related to COVID-19, such as fever, headache, cough, sore throat, breathing difficulty, loss of sense of smell, muscle aches, and chills. Respondents were asked to attribute reported deaths to a few broad categories, including stroke or heart disease (combined here because they are often confused in rural Bangladesh), cancer, liver, or lung disease. A total of 76 deaths, often in the case of an elderly parent, were reported more than once by different households and required an additional telephone call to avoid duplication.

### Economic Impact and Mobility Data

To determine the economic impacts associated with COVID-19 in the study villages, a randomly selected 20% of 16 054 households in the first telephone survey and the same 20% with an additional 8% of households in the second telephone survey were asked additional questions. A total of 2608 households participated in the first economic impact survey and 3151 households in the second.

Data from Google COVID-19 Community Mobility Reports were used to assess the outcomes associated with the government’s measures to restrict movement as a way of managing the pandemic.^18^ These reports are based on aggregated, anonymized sets of data from users who have turned on the Location History setting. The reports chart movement trends over time by geography, across different categories of places compared with a baseline. The baseline is the median value, for the corresponding day of the week, during the 5-week period January 3 to February 6, 2020. We selected transit stations and workplaces as 2 indicators among several others showing broadly similar trends.

### Statistical Analysis

#### Multilevel Regression Model of Excess Mortality and Poststratification

We estimated mortality using a multilevel regression model and poststratification to adjust for nonresponse in first, second, or both census rounds and to understand variations across demographic groups. Only mortality data obtained directly from a household rather than from neighbors who know the household were used in this analysis. The underlying assumption is that nonresponse is a function of age, sex, and education. To estimate aggregate mortality, we therefore first estimated mortality for various age, sex, and education groups and then calculated a weighted sum. We stratified observed death counts into cells according to 4 dimensions: (1) months from January 2019 through October 2020, (2) age during the evaluation month in bins of 10 years, (3) sex, and (4) household education level. We mostly considered February 2020 as the initial month of the impact of COVID-19 but also considered later months.

We set up a multilevel logistic regression^19,20^ to model baseline mortality in each stratum by a 4-way interaction of month of year, age, sex, and education (eAppendix in the Supplement). To quantify the stratum-specific excess mortality for a given month, we set another layer of multilevel model, where the monthly excess mortality was decomposed by a 3-way interaction of age, sex, and education. We completed the model with a lag-1 autoregressive prior on all age effects and month effects, and weakly informative priors on other coefficients. We performed fully bayesian inference for our complete model in Stan software^21^ using 4 chains and 3000 posterior simulation draws for each chain. Computational diagnostics indicated that all chains mixed well.

To aggregate the stratum-specific mortality to the population of interest, we generated posterior simulation draws of mortality in each stratum (eAppendix in the Supplement). Under the assumption that stratum-specific mortality rates are not affected by attrition in later rounds of census survey, the aggregate mortality is a weighted sum of mortality in each stratum, where weights are constructed using the January 2020 census data, from which we computed the excess mortality in the population by the Monte Carlo method. The poststratified excess mortality implicitly compares average mortality in 2020 with the same period in 2019. We repeated the same process to calculate the change in population-aggregated age-specific excess mortality.

#### Bayesian Model

We adopted a full bayesian model and represented uncertainty of our estimate using 95% CIs computed from Markov chain Monte Carlo draw. We fit the bayesian model in Stan version 2021 (Stan Development Team). Data analysis was performed from February to April 2021.

## Results

### Raw Data

Considering first only the 11 256 households that could each be reached during both telephone surveys, a total of 639 deaths were reported between January 2019 and the end of October 2020 for a total population of 58 806 individuals (29 726 female participants [50.5%]; mean [SD] age, 26.4 [19.8] years), excluding individuals who joined the household after the January 2020 census. This corresponds to an average annualized mortality rate of 5.9 deaths per 1000 individuals over 22 months (adjusted mortality rate, 6.1 deaths per 1000 individuals). For the same households that could be reached twice over the telephone, a total of 276 deaths were reported between February and the end of October 2020, slightly below the 289 deaths reported for over the same months in 2019.

**Figure 2.**
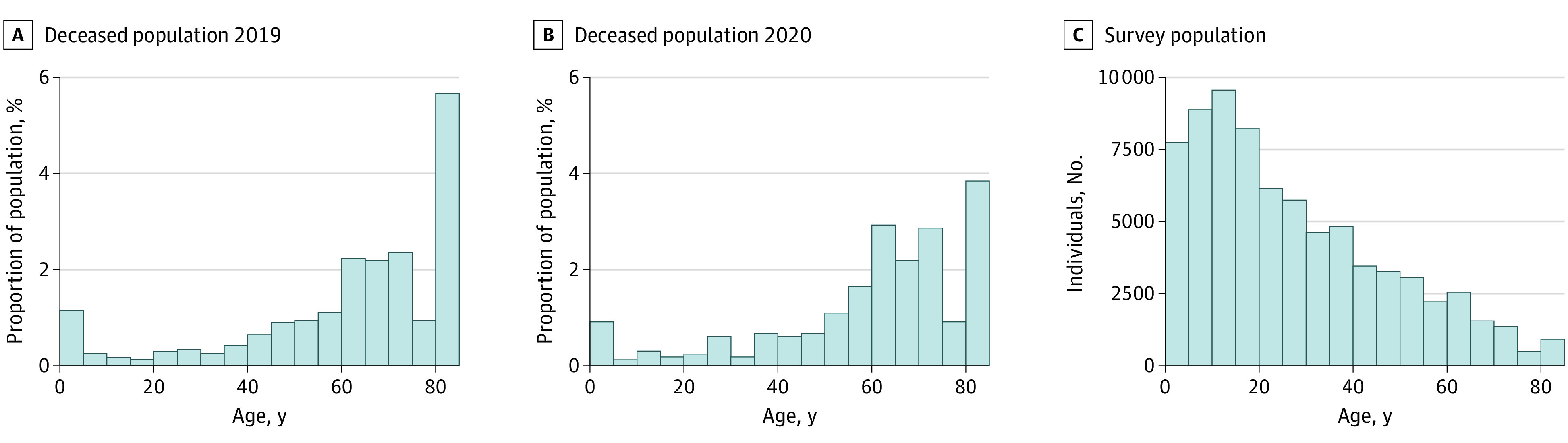
Distribution of Age at Death in Study Households Histograms show ages of individuals who died in 2019 (A) and 2020 (B) and for the entire survey (C). Reported ages above 80 years are combined.

The age distribution of the surveyed population and associated mortality rates in our data were comparable to published national trends (Figure 2).^9^ Our data did not show a clear seasonal pattern in mortality^22^ or an increase in mortality that coincides with the peak in COVID-19 cases and deaths reported centered on late May 2020 (Figure 1). The challenge is to determine whether mortality remained unaffected by the pandemic after taking into account all the available data, including households that could not be reached after the in-person census.

### Model-Based Inference of Excess Mortality

**Figure 3.**
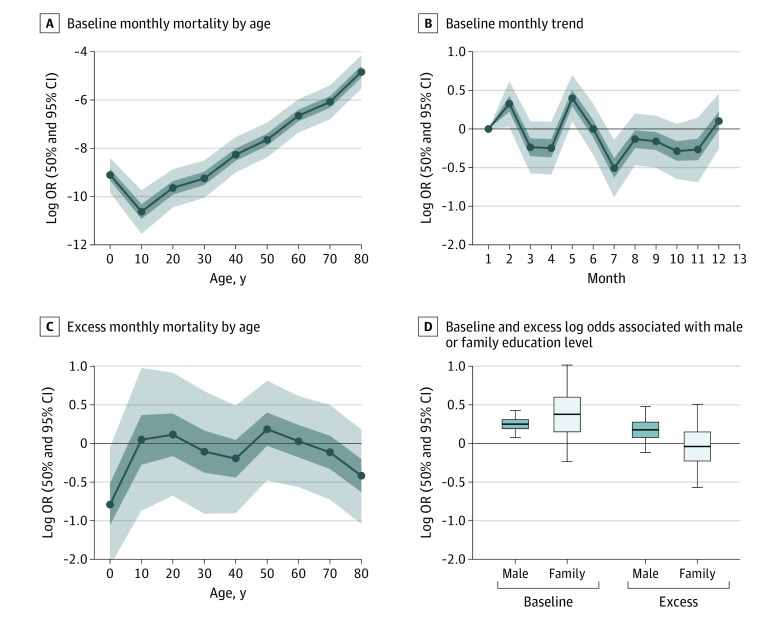
Decomposition of All Modeled Factors to Baseline and Excess Mortality in Log Odds Ratios (ORs), Visualized by the Posterior Mean, 50% CIs, and 95% CIs A, The age-specific baseline monthly mortality log OR is shown across ages. B, The baseline month-of-year effect of the monthly mortality log OR across months is shown. C, The age-specific excess monthly mortality during February to October 2020 is shown. In panels A, B, and C, dots denote log ORs, and shaded areas denote 50% CIs (darker shading) and 95% CIs (lighter shading). D, The baseline and excess log odds associated with male or family education level are shown. Lines within boxes denote means, tops and bottoms of boxes denote 50% CIs, and error bars denote 95% CIs.

The model confirmed the expected increase in mortality as a function of age and the lack of a clear seasonal pattern in mortality (Figure 3A and 3B). The posterior mean of the annualized baseline mortality rates inferred from the model range from a minimum of 0.5 deaths per 1000 individuals for the 10 to 19 years age range to a maximum of 150 deaths per 1000 individuals for age 80 years. Excess mortality was the key output from the model. At first, we assumed that the onset of the pandemic in the study villages was in February 2020. The model showed no excess mortality during February to October 2020 and, if anything, possibly a slightly lower mortality log odds ratio at the lowest and highest ages (Figure 3C). The model also suggests that excess mortality was likely higher for men compared with women across the entire range of ages (Figure 3D).

**Figure 4.**
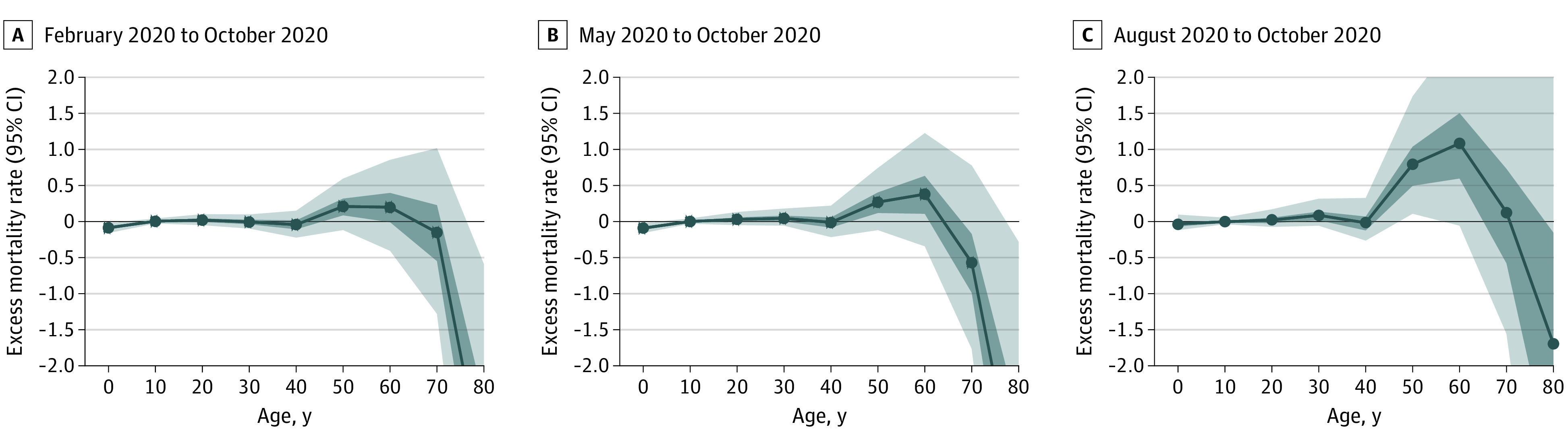
Age-Specific Mean Excess Monthly Mortality Rates and 50% CIs and 95% CIs The comparison window always ends in October 2020 whereas the starting month varies from February 2020 (A) to May 2020 (B), and August 2020 (C).

After poststratification across sex and education, the model showed no indication of an increase in mortality rate during February to October for 2020 compared with 2019 in all age groups (Figure 4A). The inferred mortality change of −8% (95% CI, −21% to 7%) was largely due to a decline in mortality in the group aged 80 years or older. Excess mortality did not vary much with household education level (a proxy for socioeconomic level) and sex (Figure 3D). Excess mortality increased to a limited extent as the boundary between the 2 comparison windows was shifted to later in 2020 (Figure 4B and 4C).

The circumstances and causes of a total of 795 deaths reported for 2019 to 2020 during the 2 rounds of telephone calls did not vary much over time. In 2019 (467 deaths) and 2020 (328 deaths), respectively, 339 (73%) and 240 (73%) deaths were preceded by consultation with a doctor or nurse, 81 (17%) and 66 (20%) deaths occurred at a hospital, and 32 (6.8%) and 28 (8.5%) deaths were the result of injury. Heart disease and stroke combined reportedly caused 192 (41%) and 154 (47%) deaths in 2019 and 2020, respectively, and cancer reportedly caused 42 (9.0%) and 30 (9.1%) deaths, respectively. The proportion of deaths attributed to lung disease actually decreased from 30 (6.4%) in 2019 to 12 (3.7%) in 2020. Among the 30 deaths attributed to lung disease in 2019, COVID-19–related symptoms such as fever, headache, cough, sore throat, breathing difficulty, loss of sense of smell, muscle aches, and chills were reported 64 times. Among the 12 deaths attributed to lung disease in 2020, the same symptoms were reported 30 times.

### Economic Impacts

**Figure 5.**
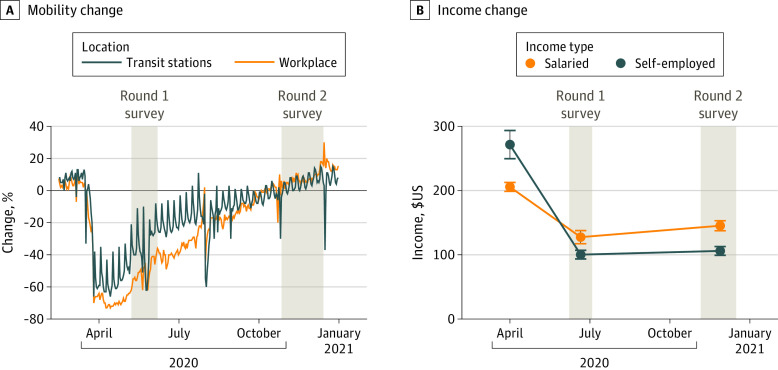
Variations in Mobility and Income Over the Study Period A, Mobility change is shown as a percentage provided by Google COVID-19 Community Mobility Reports. The baseline mobility is the median of mobility index from January 3 to February 6, 2020. B, Income change was determined from self-reported income from salaried or self-employed primary income earners from before COVID to November 2020 (the mean exchange rate for 2020 was US $1 = 84.9 Bangladeshi Taka).

Restrictions on travel and business imposed by the Bangladesh government to limit the impact of COVID-19 were associated with considerable economic impacts on our study population (Figure 5B). Approximately one-half of the primary income earners were salaried according to our data, and their monthly income decreased by 40%, from 17 485 Bangladeshi Taka (BDT) (US $206) during normal times to 10 835 BDT (US $128) in May 2020 (mean exchange rate for 2020, US $1 = 84.9 BDT). The pre-COVID-19 income was consistent with the inflation-adjusted mean rural household income of 16 597 BDT (US $196) in 2020.^16,23^ For households where the primary income earner was self-employed in May 2020, the mean monthly income was 8521 BDT (US $100) and was reduced sharply by 60% from the normal level of 23 083 BDT (US $272). In November, the mean salaried and self-employed income only partially recovered to 12 346 BDT (US $145) and 9011 BDT (US $106), respectively. Among 2768 randomly selected households, 683 (25%) reported that they could not obtain an essential food item in May 2020 because of reduced income, although this proportion had declined to 8.9% (292 of 3289 households) by November 2020. Even when the mobility of people reverted to the prepandemic levels (Figure 5A), the negative economic impact associated with the pandemic was only partially reversed.

## Discussion

The annualized adjusted mortality rate of 6.1 per 1000 calculated for the 22 months spanned by our study is higher than national estimates for rural Bangladesh in 2019 of 5.4 deaths per 1000.^9^ The difference is entirely plausible given the range of district-level mortality rates across the country. The posterior mean of the annualized baseline mortality rates inferred from the model range from a minimum of 0.5 deaths per 1000 individuals for the 10 to 19 years age range to a maximum of 150 deaths per 1000 individuals for age 80 years and older, also in agreement with national statistics (Figure 3A).^9^

On the basis of our census data collected on 3 occasions, once in person and twice over the telephone, we conclude that mortality did not increase in 2020 across our 135 study villages or *paras*. In fact, our best estimate is that mortality declined by 8%. This decrease in mortality does not appear to be associated with a decline in mortality from other causes such as road collision injuries or the seasonal influenza caused by reduced travel and social interactions, respectively. On average, the net impact of COVID-19, therefore, did not come close to the levels of excess mortality of 20% and over in 2020 reported for over 2 dozen countries including the US.^2^ Our data could suggest that most of the reported COVID-19 mortality was limited to urban areas (eFigure 1 in the Supplement), which account for approximately one-third of the Bangladesh population. Government sample vital statistics data released after this study was completed indeed indicate that urban mortality increased by approximately 10% in 2020 compared with previous years,^24^ whereas rural mortality decreased slightly (eFigure 3 in the Supplement).

Various hypotheses have been proposed to explain the apparently limited impact of COVID-19 in some low-income countries in 2020.^25^ The young population is consistent with low rural mortality but cannot explain a decrease in age-specific mortality for the older segments of the study population (Figure 4). Spending more time outside or in well-ventilated houses has been proposed as an explanation, but other factors could have dampened the symptoms of COVID-19.^26^

Google mobility data and our own economic impact data both indicate that the reach of the government’s interventions extended to rural areas of Bangladesh (Figure 5A). Our mortality data suggest that limiting gatherings, encouraging masks, and maintaining social distance may have limited the pandemic in rural areas in 2020.^27^ At the same time, measures restricting work, trade, and travel clearly imposed an economic burden on rural households that extended over at least 6 months, as reported also in a neighboring area.^28^ There is therefore a critical need for the government to expand its social safety net to the population affected by nonpharmaceutical interventions to limit the COVID-19 pandemic.^7,29^

### Limitations

We do not claim that our sample of 135 villages is necessarily representative of all of rural Bangladesh, although key demographic data are comparable to those of the rest of the county (eTable 1 in the Supplement). One limitation is that one-quarter of the households surveyed in January 2020 could not be reached over the telephone by November 2020. Our approach to calculate aggregate mortality using demographic group-level mortality corrects for this, but only to the extent that mortality estimated for a particular age, sex, and education group is not biased by nonresponse. The repeated census approach that was followed may also not have entirely eliminated a tendency not to report a recent death associated with COVID-19 symptoms because of stigma, especially at the beginning of the pandemic.^30^ We also cannot exclude that households with a member who recently died may be less likely to pick up the telephone or less willing to participate in a survey. Single-member households are an extreme case of such a situation but probably contributed no more than 4 deaths that were not counted on the basis of their age distribution (eTable 3, eTable 4, and eFigure 4 in the Supplement).

## Conclusions

For reasons that deserve further study, it does not appear that COVID-19 associated with mortality in rural Bangladesh in 2020 as much as in many other countries. Although this finding suggests that restrictions imposed by the government may have had the desired effect, our study also highlights the need for corrective public programs to address the very high economic burden resulting from such measures. The situation may be quite different in 2021 because of the Delta variant of the coronavirus.

## Notes

Supplement. **eFigure 1.** Map of Bangladesh Showing the Distribution of Confirmed COVID-19 Cases as of December 15, 2020 **eFigure 2.** Flow Chart Showing the Number of Households Covered by Various Surveys **eFigure 3.** Time Series of Rural and Urban Mortality Rates From the Bangladesh Bureau of Statistics Based on a Nationally Representative Sample Population of 1.5 Million **eFigure 4.** Histograms of Ages of Single-Member Households and Other Households in the Study **eTable 1.** Socioeconomic Statistics in Rural Bangladesh Nationwide From the 2016 Household Income and Expenditure Survey Distributed Across 2,204 Unions (BBS 2016) and in Our Sample Survey **eTable 2.** Listing of Number of Household Interviews Conducted Following AAPOR (2016) Nomenclature **eTable 3.** Proportion of Single-Member Households Covered by Different Surveys **eTable 4.** Reported Deaths for Single-Member Households in Relation to Rest of Study Population **eAppendix.** Supplemental Methods
