# Supplementary material for: Assessment of Excess Mortality and Household Income in Rural Bangladesh During the COVID-19 Pandemic in 2020
Source: JAMA Netw Open. 2021 Nov 15;4(11):e2132777. doi: 10.1001/jamanetworkopen.2021.32777 (PMC8593765; doi:10.1001/jamanetworkopen.2021.32777)
Supplement: Supplement. — eFigure 1. Map of Bangladesh Showing the Distribution of Confirmed COVID-19 Cases as of December 15, 2020 eFigure 2. Flow Chart Showing the Number of Households Covered by Various Surveys eFigure 3. Time Series of Rural and Urban Mortality Rates From the Bangladesh Bureau of Statistics Based on a Nationally Representative Sample Population of 1.5 Million eFigure 4. Histograms of Ages of Single-Member Households and Other Households in the Study eTable 1. Socioeconomic Statistics in Rural Bangladesh Nationwide From the 2016 Household Income and Expenditure Survey Distributed Across 2,204 Unions (BBS 2016) and in Our Sample Survey eTable 2. Listing of Number of Household Interviews Conducted Following AAPOR (2016) Nomenclature eTable 3. Proportion of Single-Member Households Covered by Different Surveys eTable 4. Reported Deaths for Single-Member Households in Relation to Rest of Study Population eAppendix. Supplemental Methods [file jamanetwopen-e2132777-s001.pdf]

Supplemental Online Content

Barnwal P, Yao Y, Wang Y, et al. Assessment of excess mortality and household income in rural Bangladesh during the COVID-19 pandemic in 2020. *JAMA Netw Open*. 2021;4(11):e2132777. doi:10.1001/jamanetworkopen.2021.32777

eFigure 1. Map of Bangladesh Showing the Distribution of Confirmed COVID-19 Cases as of December 15, 2020
eFigure 2. Flow Chart Showing the Number of Households Covered by Various Surveys
eFigure 3. Time Series of Rural and Urban Mortality Rates From the Bangladesh Bureau of Statistics Based on a Nationally Representative Sample Population of 1.5 Million
eFigure 4. Histograms of Ages of Single-Member Households and Other Households in the Study
eTable 1. Socioeconomic Statistics in Rural Bangladesh Nationwide From the 2016 Household Income and Expenditure Survey Distributed Across 2,204 Unions (BBS 2016) and in Our Sample Survey
eTable 2. Listing of Number of Household Interviews Conducted Following AAPOR (2016) Nomenclature
eTable 3. Proportion of Single-Member Households Covered by Different Surveys
eTable 4. Reported Deaths for Single-Member Households in Relation to Rest of Study Population
eAppendix. Supplemental Methods

This supplemental material has been provided by the authors to give readers additional information about their work.

© 2021 Barnwal P et al. *JAMA Network Open.*

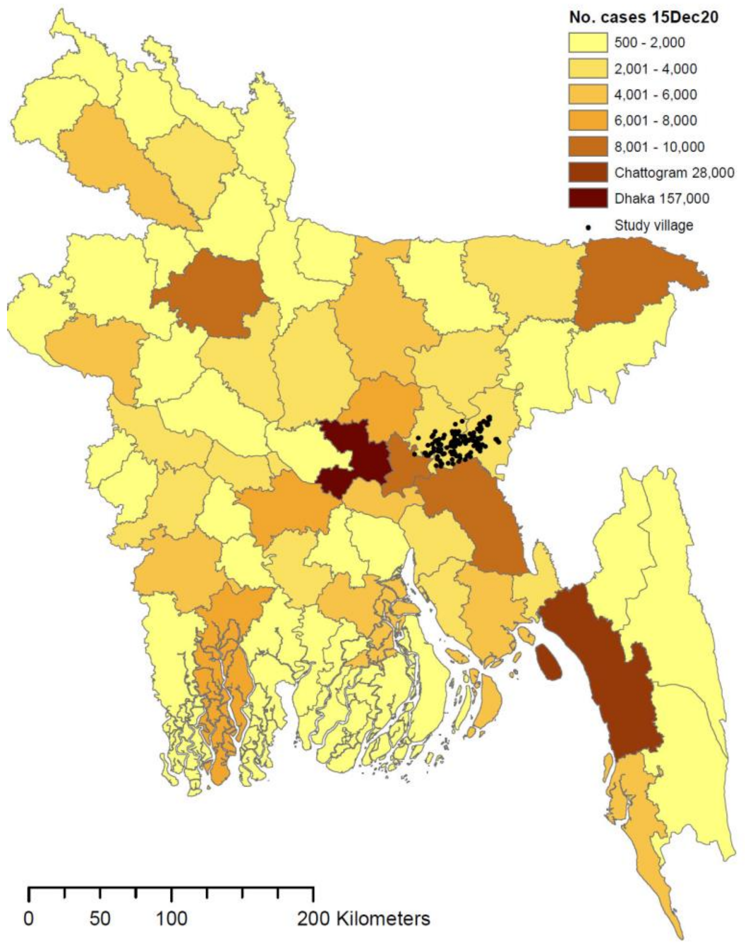

eFigure 1. Map of Bangladesh Showing the Distribution of Confirmed COVID-19 Cases as of December 15, 2020. Study villages surveyed in person and over the phone in 2020 are shown as black dots east of the capital Dhaka. In the region selected for the original study concerning well-water arsenic, the local geology allows households with a contaminated well to respond by installing a somewhat deeper private well instead of relying on a government-installed and even deeper community well (Jamil et al., *Env. Sci. Technol.* 2019). We targeted villages of intermediate size based on available census data and Google Earth imagery but often had to narrow the study to portions of villages to avoid having to test too many wells.

© 2021 Barnwal P et al. *JAMA Network Open.*

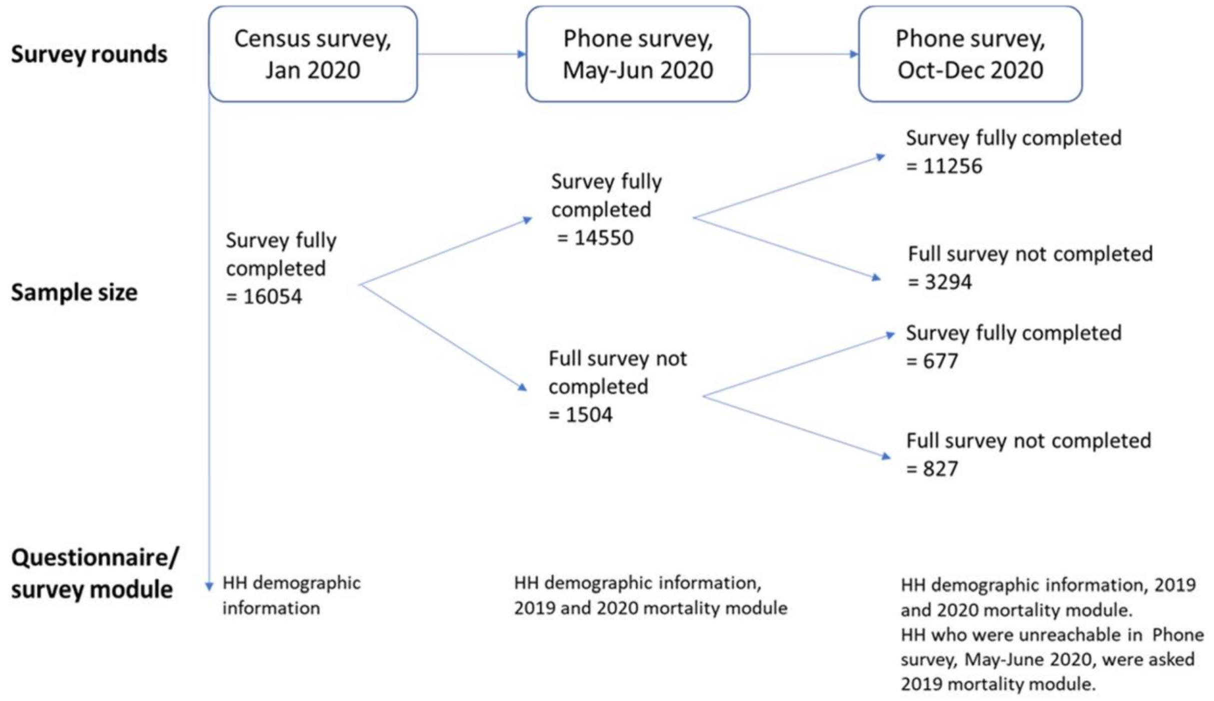

eFigure 2. Flow Chart Showing the Number of Households Covered by Various Surveys. At the beginning of a phone call, each consenting respondent was asked to list current household members without prompting, after which discrepancies where investigated. Only at the end of the first phone call were respondents asked directly about any deaths that occurred in the household in 2019. The surveys involved40-60 enumerators working in the field for two weeks and later twice over the phone for at least a month.

© 2021 Barnwal P et al. *JAMA Network Open.*

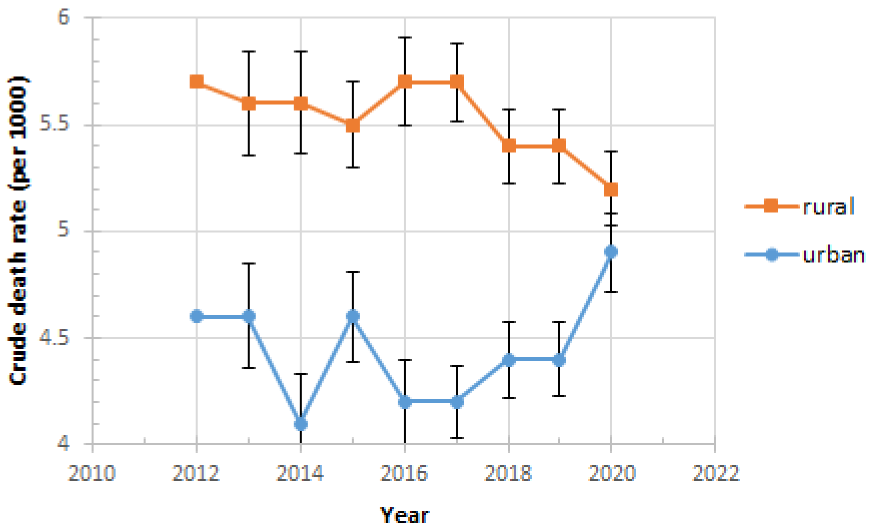

eFigure 3. Time Series of Rural and Urban Mortality Rates From the Bangladesh Bureau of Statistics Based on a Nationally Representative Sample Population of 1.5 Million. Two-sigma error bars based on the proportion of deaths and the sample size could be readily calculated only for the most recent two years. Data from http://www.bbs.gov.bd/site/page/b588b454-0f88-4679-bf20-90e06dc1d10b/-

© 2021 Barnwal P et al. *JAMA Network Open.*

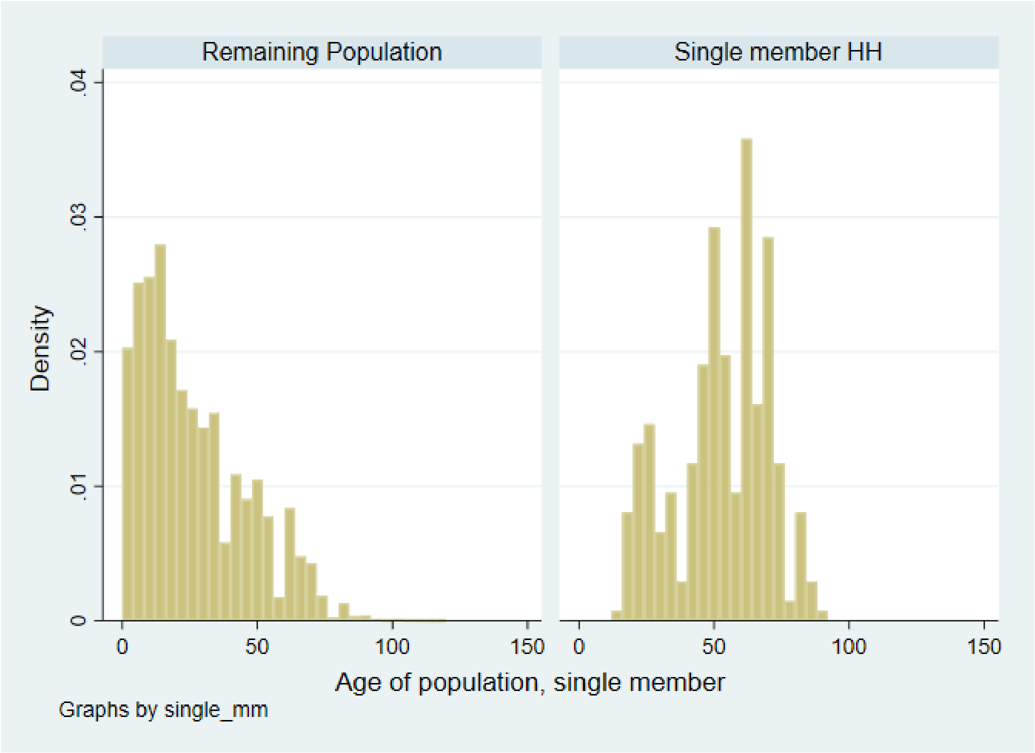

eFigure 4. Histograms of Ages of Single-Member Households and Other Households in the Study.

© 2021 Barnwal P et al. *JAMA Network Open.*

eTable 1. Socioeconomic Statistics in Rural Bangladesh Nationwide From the 2016 Household Income and Expenditure Survey Distributed Across 2,204 Unions (BBS 2016) and in Our Sample Survey.

Variable	Unions in rural Bangladesh, HIES 2016			Unions in this study, HIES 2016			NSF Survey, 2020		
	N	Mean	SD	N	Mean	SD	N	Mean	SD
Age	129,026	28	19.8	1,393	25	19.3	80,556	26	19.6
Sex	129,027	0.50	0.50	1,393	0.51	0.50	80,556	0.51	0.50
Rooms in house	31,748	2.32	1.18	299	2.31	1.08	12,286	2.71	1.31
Formal education	31,783	0.85	0.36	300	0.85	0.36	16,052	0.89	0.32
Primary education	31,795	0.29	0.28	300	0.23	0.25	16,054	0.29	0.26
Agriculture occup.	31,795	0.18	0.23	300	0.08	0.14	16,054	0.08	0.13
Household size	31,795	4.06	1.57	300	4.65	1.64	16,054	5.02	2.03
Male ratio	31,795	0.49	0.19	300	0.48	0.20	16,054	0.48	0.19
Child ratio	31,795	0.34	0.22	300	0.43	0.22	16,054	0.39	0.21
Sex household head	31,651	0.13	0.33	289	0.18	0.39	16,054	0.27	0.44
Age household head	31,579	45	14.3	289	44	14.4	16,039	45	14.5

The number of observation corresponds to individuals age (years) and sex (Female=1, Male=0) and to household for count of rooms, formal education (1=over half of the household received any formal education), primary education ratio (proportion of household member who completed primary education), agricultural activity proportion of household member engaged in agricultural activity), count of household member, male ratio, child ratio, household head's sex (Female=1, Male=0), household head's age. Household head's education (0= none, 1=class 1, 2=class 2, 3=class 3, 4=class 4, 5=class 5/PSC, 6=class 6, 7=class 7, 8=class 8, 9=class 9, 10=SSC/Dakhil, 11=HSC/Alim first year, 12= HSC/Alim 2nd year, 13=BA/BSC/Fazil 1st year, 14=BA/BSC/Fazil 2nd year, 15=BA/BSC/Fazil 3rd year, 16=BA/BSC/Fazil 4th year, 17=MA/MSC/Kamil and above).

© 2021 Barnwal P et al. *JAMA Network Open.*

eTable 2. Listing of Number of Household Interviews Conducted Following AAPOR (2016) Nomenclature.

Variable	Census Survey Jan-Feb 2020	Phone Survey May-Jun 2020	Phone Survey Oct-Nov 2020
I = Complete interview (1.1)	16,054	14,550	11,933
P = Partial interview (1.2)		1160	2459
R = Refusal and break-off (2.10)	6	216	1117
NC = Non-contact (2.20)	1478	4	1
O = Other (2.30)			
UH = Unknown if household/occupied HU (3.10)		103	477
UO = Unknown, other (3.20, 3.30, 3.40, 3.90)		21	67
e = Estimated proportion of cases of unknown eligibility that are eligible			
Total	17,538	16,054	16,054
RR1	0.9154	0.9063	0.7433
RR2	0.9154	0.9786	0.8965
COOP1	0.9996	0.9136	0.7694
REF1	0.0003	0.0135	0.0696
CON1	0.9157	0.9920	0.9661

Response rates:
RR1 = I/(I+P)+(R+NC+O)+(UH+UO)
RR2 = (I+P)/(I+P)+R+NC+O)+(UH+UO)
Corporation rate: COOP1 = I/(I+P)+R+O
Refusal rate: REF1 = R/(I+P)+(R+NC+O)+(UH+UO)
Contact rate: CON1 = (I+P)+R+O/(I+P)+R+O+NC+(UH+UO)

Here, P includes neighbor responses concerning deaths.
For the Census survey, NC= Count of absent HHs+ count of temporarily and permanently migrated HHs+ Count of HH where eligible respondents were not found.
For phone surveys, NC= Count of HHs where the respondent wasn't available during the survey period.
UH= Count of HH where no one picked up+ phone was switched off
UO = Count of HH where census contact number reported as wrong number.

© 2021 Barnwal P et al. *JAMA Network Open.*

eTable 3. Proportion of Single-Member Households Covered by Different Surveys. Of potential concern is that the proportion of single-member households that could be reached declined from 2.1% during the in-person survey to 1.8 and 1.6% during the first and second phone survey, respectively.

	Census survey, Jan 2020	Phone survey, May-Jun 2020	Phone survey, Oct-Dec 2020
Single member HH	342 (2.1%)	261 (1.8%)	189 (1.6%)
>1 member HH	15,712	14,289	11,744
Total	16,054	14,550	11,933

© 2021 Barnwal P et al. *JAMA Network Open.*

eTable 4. Reported Deaths for Single-Member Households in Relation to Rest of Study Population.

Each Phone survey data was collected in two phases. In Phase I, we called every HH for two days (with one day gap between). In Phase II, we try to reach the HH in following category – phone switched off, phone rang but no one picked up, wrong number – through their neighbors (up to 3 closest neighbor, whom we already surveyed in Phase I). We called every neighbor and asked whether they recognize the HH and share any referral contact number. Also asked if they can tell us whether any one in that HH died in 2019 and 2020 and some addition information related to deceased person.

Survey response, Phone survey Oct-Dec 2020	HH count, as per Census Jan 2020	2020 Reported deaths by the HH (overall)	2020 Reported deaths by neighbor 1	2020 Reported deaths by neighbor 2
a) Reached single-member HHs	189	7		
b) Unreached single-member HHs	153			
b.1) HH known to neighbor	80		1	1
b.2) HH not known to neighbor	31			
b.3) HH response: No consent	29[1]	1		
b.4) HH response: Refused to talk	9			
b.5) HH response: Partially completed	4[2]			
a) Reached multi-member HHs	11,744	293		
b) Unreached multi-member HHs	3,968			
b.1) HH known to neighbor	1,944	16	17	12
b.2) HH not known to neighbor	559	3		
b.3) HH response: No consent	659	10		
b.4) HH response: Refused to talk	375	5		
b.5) HH response: Partially completed	431	8		
**Total**		**343**		

Note: [1]In 24 non-consent HH, enumerator spoke to the only listed member during phone survey October-December 2020. So, they were alive. In remaining 5 HH, other HH member, who weren't listed during the census survey, answered the call.

[2]These 4 HHs completed the morality module and reported no deaths in 2020.

Using age-specific death rates calculated from our data, however, we estimate that approximately 3.9 deaths (95% confidence interval 1.7–7.2) may not have been accounted for because the single member of that household could not be reached. This number is equal to 0.5% of the total number of reported deaths. For further confirmation, we called up to two neighbors of the 153 single-member-households that could not be reached directly. Neighbors who indicate that they knew 80 single-member households reported 1 additional case of death, which amounts to 4.3 estimated total deaths among the two of 342 single-member households and is very close to the separate estimate based on age-specific death rates.

© 2021 Barnwal P et al. *JAMA Network Open.*

eAppendix. Supplemental Methods

Baseline mortality rate. We use the following notation for the four discrete variables: month j_1 = 1, . . . , 22; age during the evaluation month, j_2 = 1, . . . , 9; gender j_3 = 1, 2; and household education j_4 = 1, 2. Any individual stratum can be written as $j = (j_1, j_2, j_3, j_4)$. In each stratum j, we count n_j the number of surveyed individuals that were alive in the beginning of the month and accessible throughout the month, and y_j the number of deceased individuals. Assuming independent sampling, the data model is

$$y_j \sim \text{Binomial}(n_j, 0_j),$$

where the parameter 0_j is what we want to estimate: the monthly mortality rate in month j_1 for age group j_2, gender j_3, and household education level j_4.

The baseline ($j_1 < t_{\text{start}}$) mortality rate for stratum j is modeled as a function of month and individual's age-sex-education attributes,

$$0_j = \text{logit}^{-1}\ a_{[j_1 \bmod 12]} + \ _{[j_2]} + \ \ j_3 = 2\ \ _{\text{male}} + \ \ j_4 = 2\ \ _{\text{edu}}\ ,\ \forall j_1 < t_{\text{start}}. \quad (1)$$

where the free parameters are

- The month-of-year factor, or the seasonal trend, denoted by $a_1, \ldots, a_{12}$ for January to December each year. Due to periodicity, cell j processes this seasonal factor $a_{[j_1 \bmod 12]} \in$ R. For identification, we set January as baseline such that $a_1 = 0$.
- The age factor, denoted by $_1, \ldots, \ _9$ for 9 age categories. The age factor for cell j is $_{[j_2]} \in$ R.
- Th e male factor $_{\text{male}} \in$ R. We set female as reference, then the sex factor for cell j is $j_3 = 2\ \ _{\text{male}}$.
- The education factor $_{\text{edu}} \in$ R. We set non-education as reference, and the education factor for cell j is $j_4 = 2\ \ _{\text{edu}}$.

Mortality in 2020. We take a flexible approach by specifying a comparison-starting month t_{start}, which splits time into the two periods (a) the baseline: Jan 2019, Feb 2019, . . . , $t_{\text{start}} - 1$, and (b) the potential excess period: $t_{\text{start}}, t_{\text{start}} + 1, \ldots$, Oct 2020. During the second period, on top of the baseline model (1), we model the stratum-specific excess risk:

$$0_j = \text{logit}^{-1}\ a_{[j_1 \bmod 12]} + \ _{[j_2]} + \ \ j_3 = 2\ \ _{\text{male}} + \ \ j_4 = 2\ \ _{\text{edu}} + \ ^{\text{excess}}_{[j_2]} + \ \ j_3 = 2\ \ ^{\text{excess}}_{\text{male}} + \ \ j_4 = 2\ \ ^{\text{excess}}_{\text{edu}}\ ,\ \forall j_1 \geq t_{\text{start}}, \quad (2)$$

where additional parameters ^{excess}, $^{\text{excess}}_{\text{male}}$ and $^{\text{excess}}_{\text{edu}}$ represent the excess risk in 2020 associated in the age, sex, and education, on top of the baseline risk.

© 2021 Barnwal P et al. *JAMA Network Open.*

Partial-pooling and priors. We complete the model by weakly-informative priors:

$$a \; - a_{-1} \sim \text{normal}(0, \;_1), \quad = 2, \ldots, 12; \; a_1 - a_{12} \sim \text{normal}(0, \;_1), \quad _1 \sim \text{normal}^+(0, 1),$$

$$- \quad _{-1} \sim \text{normal}(0, \;_2), \quad = 2, \ldots, 9, \quad _2 \sim \text{normal}^+(0, 1), \quad _1 \sim \text{normal}(-8, 4),$$

$$^{\text{excess}} - \; ^{\text{excess}}_{-1} \sim \text{normal}(0, \;_3), \quad = 2, \ldots, 9, \quad _3 \sim \text{normal}^+(0, 1), \quad ^{\text{excess}}_{1} \sim \text{normal}(0, 1),$$

$$^{\text{excess}}_{\text{male}}, \; ^{\text{excess}}_{\text{edu}}, \; _{\text{male}}, \; _{\text{edu}} \sim \text{normal}(0, 1).$$

Aggregate mortality in the population. After model is fitted using Markov chain Monte Carlo simulations, we generate posterior simulations of the demographic-specific baseline mortality :

$$\tilde{0}_{j_2,j_3,j_4} = \text{logit}^{-1} \Big(\; _{[j_2]} + \; _{j_3 = 2} \; _{\text{male}} + \; _{j_4 = 2} \; _{\text{edu}} + \frac{1}{12} \sum_{j_1=1}^{12} a_{j_1} \Big),$$

and the demographic-specific mortality in 2020 after t_{start}:

$$\tilde{0}^*_{j_2,j_3,j_4} = \text{logit}^{-1} \Big(\; _{[j_2]} + \; _{j_3 = 2} \; _{\text{male}} + \; _{j_4 = 2} \; _{\text{edu}} +$$

$$^{\text{excess}}_{[j_2]} + \; _{j_3 = 2} \; ^{\text{excess}}_{\text{male}} + \; _{j_4 = 2} \; ^{\text{excess}}_{\text{edu}} + \frac{1}{12} \sum_{j_1=1}^{12} a_{j_1} \Big).$$

We use census data to construct the demographic post-stratification weight w_{j_2,j_2,j_3}: the proportion of individuals in our round-1 survey with age j_2, gender j_2 and educational level j_4. Then the monthly excess mortality of any given age group j_2 is computed by

$$\text{excess mortality}\,[j_2] = \frac{\sum_{j_3,j_4} w_{j_2,j_3,j_4} (\tilde{0}^*_{j_2,j_3,j_4} - \tilde{0}_{j_2,j_3,j_4})}{\sum_{j_3,j_4} w_{j_2,j_4,j_4}}. \tag{3}$$

Likewise, we aggregate this excess mortality across all ages in the population:

$$\text{excess mortality}_{\text{all age}} = \frac{\sum_{j_2,j_3,j_4} w_{j_2,j_3,j_4} (\tilde{0}^*_{j_2,j_3,j_4} - \tilde{0}_{j_2,j_3,j_4})}{\sum_{j_2,j_3,j_4} w_{j_2,j_4,j_4}}. \tag{4}$$

To compute the relative mortality change, we divide this excess rate by the average baseline mortality,

$$\text{excess mortality}_{\text{relative, all age}} = \text{excess mortality}_{\text{all age}} \times \frac{\sum_{j_2,j_3,j_4} w_{j_2,j_4,j_4}}{\sum_{j_2,j_3,j_4} w_{j_2,j_3,j_4} \tilde{0}_{j_2,j_3,j_4}} \times 100\%. \tag{5}$$

© 2021 Barnwal P et al. *JAMA Network Open.*

Census In-Person Survey Jan-Feb 2020

Survey Questionnaire/ Relevant module

Section 1: Survey Tracking Sheet

<table>
<tr><td colspan="4">Identification: 1a. Household ID: [____]</td></tr>
<tr><td colspan="2">2a. Enumerator ID: [____|____|____]</td><td colspan="2">2b. Enumerator Name: [___________]</td></tr>
<tr><td colspan="4">3. Location of Enumeration Area:
Bangladesh
a. Division (use R2b codes): [____|____|____]
b. District (use R3b codes): [____|____|____]
c. Upazila / Thana OR City Corporation (use R4b codes): [____|____|____]
d. Union OR Municipality (use R5b codes): [____|____|____]
e. Village/Moholla (use R6b codes): [____|____|____]</td></tr>
<tr><td colspan="2">4. Will the whole village be surveyed?</td><td colspan="2">Yes 1
No.. 0</td></tr>
<tr><td colspan="2">5. Para (if available)</td><td colspan="2">..</td></tr>
<tr><td colspan="2">6. Landmark</td><td colspan="2">..</td></tr>
<tr><td colspan="2">7. Is it your first day in this village?</td><td colspan="2">Yes 1
No....................................... 0</td></tr>
<tr><td colspan="2">8. If Q.4 = No,
Where does this HH locate?</td><td colspan="2">Outer region of the para 1
Within the para..................................... 2</td></tr>
<tr><td colspan="2">9. Interviewer ID:</td><td colspan="2">[___|___|___|___]</td></tr>
<tr><td colspan="4">10. Interviewer's full name: ____</td></tr>
<tr><td colspan="4">QUESTIONNAIRE VERIFICATION (for paper surveys only)</td></tr>
<tr><td></td><td>a. SUPERVISOR:</td><td>b. CHECKED BY:</td><td>c. ENTERED BY:</td></tr>
<tr><td>11. ID Code:
12. Initials:
13. Date (Gregorian dd/mm/yy):</td><td>[___|___|___]
____
[__|__]/[__|__]/[__|__]</td><td>[___|___|___]
____
[__|__]/[__|__]/[__|__]</td><td>[___|___|___]
____
[__|__]/[__|__]/[__|__]</td></tr>
<tr><td colspan="4">COMMENTS:</td></tr>
<tr><td colspan="4"></td></tr>
</table>

© 2021 Barnwal P et al. *JAMA Network Open.*

Instruction for enumerator: Please administer the survey to preferably an elder female member in the household (or to a person knowledgeable about household members).

Consent Note:

Hello, my name is...............and I am working with Innovations for Poverty Action (IPA), Bangladesh, a research and policy non-profit organization that trying to help with Rural Household facing problems. We are inviting you to take part in this survey as we considered you knowledgeable to answer a few questions about your household member and water source, which will take a maximum of 15 minutes. If you have no objection, allow IPA to take "GPS" coordinates of where we are conducting the interview. This means we will use our phone to mark our interview location on a map.With your permission, we would also like to attach a tag on your well and take well GPS coordinate. We hope that this research will help us better understand the needs of the community in order to improve future interventions directed towards arsenic mitigation.

Note that all the information collected will be treated as strictly confidential. You have the right to decline to answer any question and to stop the interview at any time. We'll also collect your name and phone number for future references but no names will be published from the study. We may re-visit your household for further data collection. You will not receive any compensation from answering the questions, however the information collected will help IPA empower the rural people in the region.

If you have any questions regarding our project or want to know in more details, kindly contact Innovations of Poverty Action through+88 01712-121221, Md. Alamgir Kabir.

Did you read the above statement aloud to a member of the household, who gave consent to the survey (or to a person knowledgeable about household members)? –

Yes ....1 >> Enumerator will continue the survey.

No……….2 >> Enumerator will not complete the whole survey. Please thank the household member for his or her time. Let the member of the household know that you will return at another time as per schedule, then complete the final steps of this survey.

Section 2.1: Respondent and household information

1. Date of interview (Gregorian dd/mm/yy):<br>Date of interview (local dd/mm/yyyy):	[___|___]/[___|___]/[___|___]<br>[___|___]/[___|___]/[___|___|___|___]
1.a. Start time (24-hour clock; hh:mm):	[___|___] : [___|___]
2.a. Respondent's Name	__________________________

© 2021 Barnwal P et al. *JAMA Network Open.*

3. Respondent's Phone Number …………………………… Alternative Phone Number………………………………..
4.How many members are there in your household? (Integer) [___|___]
Note: Section 2.2 will repeat for every household member<br>(Start with the name of the HH head, HH head's spouse, then of his/her elder son, son's wife, younger son, son's wife, then elder daughter, daughter's husband, then with the children - starting from the oldest to the youngest in this manner.)

Section 2.2: Demographic information

5.a	5.b	5.c	5.d	5.e	5.f	5.g	5.h<br>If Relationship code=1			
Name of HH member.	Relationship with household head.<br><br>Use Relationship Codes below.	Age of the member<br><br>(In Years)	Sex of the member.<br><br>1.Male<br>2.Female	Highest level of education completed<br><br>Use Education Codes at page 41.	Type of main occupation of the member<br><br>Use Occupation Type Codes age at page 41	Specify the type of main occupation the member<br><br>Use Occupation Codes at page 42	Marital status of HH head.<br><br>Use Marital Status codes at page 41.	Spouse's name of HH head.<br><br>Not applicable if unmarried. (Marital status=7))	Father's name of HH head.<br><br>If Marital status=1 or 3 or 5 or 6 or 7or 8	Father-in-law's name of HH head.<br><br>If Marital status=2 or 4
1.<br>2.<br>3.<br>4.<br>5.<br>6.<br>7.										

6. Collect HH GPS:

© 2021 Barnwal P et al. *JAMA Network Open.*

Phone Survey May-June 2020

Survey Questionnaire/ Relevant module

Section 1: Survey Tracking Sheet

**Sl**	**Question**	**Answer**
TR1	Date of interview (dd/mm/yy):	[___|___]/[___|___]/[___|___]
TR2	Start time (hh:mm; 24-hour clock):	[___|___] : [___|___]
TR3	Interviewer ID/Name (First Middle Last):	[___|___]__________________________________
TR4	Household ID/ Case ID:	[____|____|____]
TR5.1	The HH address from survey was<br>District:<br>Upazila:	 [__|__]<br>[__|__]
TR5.2	Union/Ward:	[__|__]
TR5.3	Village:	[__|__]
TR5.4	Para:	_________________
TR5.5	Landmark	_________________
TR5.6	Household head	_________________

TR 6. Phone response: Please call the household head at given contact numbers.

Instruction for enumerator: Search primarily for household head or respondent of the Census survey. See If any of them are available. First preference for selecting respondent would be female adult member. If the female adult member is busy or not available then we would prefer respondent of the Census survey. If respondent of the Census survey not found then we would prefer household head. If any children pick up the call ask, Is any of adult female member (name of household head or respondent of the Census survey available)? If yes, try to interview one of them.

© 2021 Barnwal P et al. *JAMA Network Open.*

Result of HH Search 1: [__]	Correct household and respondent found 1	

Result of HH Search 2: [__]	No interview: Respondents did not pick up.................. 2	>> Try next phone number
Result of HH Search 3: [__]	No interview: Phone was switched off 3	>> Try next phone number
Result of HH Search 4: [__]	No interview: Respondent will not be available during survey period 4	>> Try next phone number
Result of HH Search 5: [__]	No interview: Respondent available at a later time… 5	>> Reschedule
Result of HH Search 6: [__]	No interview: Refusal to talk… 6	>> Try next phone number
Result of HH Search 7: [__]	No interview: Wrong household/ Wrong number.……7	>> TR6.2

Instruction for enumerator: If given contact number is belong to a neighbor. Please ask following.

Note: We surveyed your HH earlier and calling you again this time to ask about one of neighbor.

**Sl**	**Question**	**Answer**
TR6.2	If TR6.1=7, Do you know any of {census's respondent name} ${household head name}? In Jan 2020, they lived in the location above.	Yes……1 >>TR6.3<br>No…… 0 >>Try next phone number
TR6.3	Could you please give us a number for {census's respondent name} ${household head name}?	Yes 1 >>TR6.3.1 No 0
TR6.3.1	Please share the number	[__________________________]
TR6.4	[For Enumerator. Do not read aloud]<br>If neighbor received then, did you collect following information from neighbor - about the household head's location, 2019/2020 deaths related information etc.?	Yes 1 >>Try next phone number<br>No 0

Section 2: Neighbor questions

Note for enumerator: Please ask two neighbors about household head's household location, 2019/2020 deaths related information etc.

Sl	Question	Answer
NQ1	I would like to request a few a question about household head's household that we are collecting for research purpose only.	
NQ2	Does the HH still live there?	Yes… ..1<br>No……0 >>NQ2.1
NQ2.1	Could you please tell me, where did the HH moved from here after Jan 2020?	Moved temporarily… .... 1<br>Moved outside of the village permanently................... 2<br>Moved somewhere else in the village permanent .... 3<br>All HH member died... 4<br>Other……………………………………………….-96 >>NQ2.1.1
NQ2.1.1	Please specify	
MR1	Are you aware of whether any member that HH has passed away in between Jan, 2019 to Dec, 2019? How many?<br>Write "0" If no one died. If the neighbor doesn't know, write "-99"	|________| members<br>If "0" or "-99" >> MR6

Note: I would like to request you to answer a few questions about the member died in 2019 (Jan - Dec) in that HH.

Instruction for enumerator: Ask details about member who passed away in 2019- starting from youngest to oldest - in this manner

	Question	First deceased	Second deceased	Nth deceased
MR2	Please tell me the name of the … person who died in 2019?	________________________	________________________	________________________

© 2021 Barnwal P et al. *JAMA Network Open.*

MR3	What was the age of … deceased? "Integer"	|___|___| years	|___|___| years	|___|___| years
MR4	What was the sex of … deceased?	Male………1<br>Female……2	Male………1<br>Female……2	Male………1<br>Female……2
MR5	When did … die? "Select date, month, year."	|___|____|_______| years	|___|____|_______| years	|___|____|_______| years

MR6	Are you aware of whether any member that HH has passed away in 2020? How many?<br>Write "0" If no one died. If the neighbor doesn't know, write "-99"	|________| members<br>If "0" or "-99" >> MR6

Note: I would like to request you to answer a few questions about the member died in 2020 in that HH.

Instruction for enumerator: Ask details about member who passed away in 2020- starting from youngest to oldest - in this manner

Sl	Question	First deceased	Second deceased	Nth deceased
MR7	Please tell me the name of the … person who died in 2020?	Select from the dropdown menu	Select from the dropdown menu	Select from the dropdown menu
MR8	What was the age of … deceased? "Integer"	|___|___| years	|___|___| years	|___|___| years
MR9	What was the sex of … deceased?	Male………1<br>Female……2	Male………1<br>Female……2	Male………1<br>Female……2
MR10	When did … die? "Select date, month, year."	|___|____|_______| years	|___|____|_______| years	|___|____|_______| years

MR11	Has anyone died due to Covid19 in that household in 2020?	Yes 1<br>No 2<br>Don't know -99

© 2021 Barnwal P et al. *JAMA Network Open.*

NOTE: If the correct household has been reached (i.e., if TR6.1=1), then the following questions will appear.

Section 3: Respondent information and Consent

**Sl**	**Question**	**Answer**
**RI1**	Who am I talking with?	[_________________________]<br>**Select from the drop-down menu. IF RI1=-96>>RI2**
**RI2**	Name of the respondent	[______________________________]

R14. CONSENT NOTE (for short survey):

Hello, my name is ……. and I am working with Innovations for Poverty Action (IPA), Bangladesh, a research and policy non-profit organization that trying to help with Rural Household facing problems. Previously we conducted a Census survey in your HH and collected information about HH members, water sources (also attached a tag on well). Now, we are inviting you to take part in this phone call survey as we considered you knowledgeable to answer a few questions about your household member health condition, morbidity and current pandemic. The survey will take around 10 minutes.

We hope that this research will help us better understand the mortality trend and impact of current pandemic of the community in order to improve future interventions directed towards public health improvement. Note that all the information collected will be treated as strictly confidential. You have the right to decline to answer any question and to stop the interview at any time. We may contact with your household for further data collection. The information collected will help IPA empower the rural people in the region. If you have any questions regarding our project or want to know in more details, kindly contact Innovations of Poverty Action through+88 01712-121221, Md. Alamgir Kabir.

R15. CONSENT NOTE (for long survey):

Hello, my name is ……. and I am working with Innovations for Poverty Action (IPA), Bangladesh, a research and policy non-profit organization that trying to help with Rural Household facing problems. Previously we conducted a Census survey in your HH and collected information about HH members, water sources (aslo attached a tag on well). Now, we are inviting you to take part in this phone call survey as we considered you knowledgeable to answer a few questions about your household member health condition, morbidity and current pandemic. The survey will take around 25 to 30 minutes.

© 2021 Barnwal P et al. *JAMA Network Open.*

We hope that this research will help us better understand the mortality trend and impact of current pandemic of the community in order to improve future interventions directed towards public health improvement. Note that all the information collected will be treated as strictly confidential. You have the right to decline to answer any question and to stop the interview at any time. We may contact with your household for further data collection. The information collected will help IPA empower the rural people in the region. If you have any questions regarding our project or want to know in more details, kindly contact Innovations of Poverty Action through+88 01712-121221, Md. Alamgir Kabir

© 2021 Barnwal P et al. *JAMA Network Open.*

**RI5**	Did you inform about the above statement to a member of the household who gave the consent to the survey (or to a person knowledgeable about household members)?	Yes….1<br>No…. 2 **>>RI8 and End of the survey**
**RI6**	Gender of Respondent	Male 1<br>Female 2
**RI7**	What is the relation of respondent with ${name_1}?<br>Previously we listed ${name_1} as household head. Please tell me your relation with ${name_1}	(See relationship codes page no=8)
**RI8**	You will not complete the whole survey. Please thank the household member for his or her time. Let the member of the household know that you will call another time as per schedule, then complete the final steps if this survey	
**RI9**	Do you remember talking to our team	Yes 1 **>>RI10**<br>No 0
**RI10**	How long ago was it?	[____] MONTHS

Section 4: Household roster update section

**Sl**	**Question**	**Answer**
**RU03**	Firstly, I would like you to list all current household members. Please tell me names of all current HH members.<br>Please note HH member means who<br>- frequently stays in this HH i.e., lives together and shares kitchen together.<br>- contribute financially and primary decision maker the HH or<br>- temporarily living outside of the HH but rely on HH income and regarding decision making (e.g. Student)<br>Any of the above criteria is sufficient to recognized one as a HH member.	**[DO NOT READ OUT OPTIONS LOUDLY**. Record what respondent say<br>Other: not in the list -96>>RU04<br>Name 1<br>Name 2<br>Name 3<br>Name 4
RU04	How many HH members were not in the list? "Inter number"	|_____| members

© 2021 Barnwal P et al. *JAMA Network Open.*

	If RU>=1, Collect newly arrived member's information - starting from the oldest to the youngest in this manner following Section 4.1<br><br>Newly arrived people mean who have come to the household after the listing survey or respondent might forget to mentioned his/her name before.	

Section 4.1: New household member demographic Part 1

RU05	RU06	RU07	RU08	RU09	RU10	RU10.1	RUH10.2
Sl number	Name of HH member?	Sex of HH members<br><br>1.Male<br><br>2.Female	Age of the members<br><br>(In year)	Relationship with household head<br><br>(See relationship codes at page 41)	Type of main occupation of the member<br><br>(See occupation codes at page 41)	Specify the type of main occupation of the members<br><br>(See occupation type codes at page 42)	If RU10.1=other, please specify the occupation of members
1.<br><br>2.<br><br>3.<br><br>4.							

Section 4.1: New household member demographic Part 2

RU10.3	RU1 0.4	RU10.5	RU11	RU11.1	RU12	RU13

© 2021 Barnwal P et al. *JAMA Network Open.*

If RU10=Other wage labor (specify)	If RU10= Other salaried worker(specify)	If RU10=Other self-employed (specify)	Where did the new member arrive from recently?<br><br>(See location codes at page 40)	If RU11=Other, Please Specify	When did the new member arrive in the household?	Permanent or a temporary member of your household?<br><br>Permanent 1<br><br>Temporary 2
1.<br><br>2.<br><br>3.<br><br>4.						

Section 5: Mortality section 2019

**Sl**	**Question**	**Answer**
MO01	**Note**: Now, I would like to ask you a few questions about the changes in Household composition in last year (2019) as well HH member's heath condition	
MO02	"I am sorry if this is painful to recollect but we need to know this for our study. Did any members of your household pass away in 2019?<br><br>Please note that Household member means Who eat together and live together in the house. Whoever visiting this HH for a short period of time/temporarily, won't be considered as HH member.<br><br>Household head means who recognized as the household head by all the members of the household and who normally makes all decisions of household. "	Yes 1<br><br>No 0 >>MO02.1 & move to section
MO02.1	IF MO02=0) Note: I am glad no one died in 2019.	

© 2021 Barnwal P et al. *JAMA Network Open.*

MO03	"How many members of your HH member passed away in between Jan, 2019 to Dec, 2019? ""Inter Number""<br><br>Enumerator: Ask about the household member who passed away in 2019. HH member means who -<br><br>- who consistently lived together and shared the kitchen together.<br><br>- who contributed financially or made HH decisions.<br><br>- who were temporarily outside of the HH but relied on HH income or regarding decision making. For example, Student living outside.<br><br>Any of the above criteria is sufficient to recognized one as a HH member.	
MO04	Note: Ask details about member who passed away in 2019- starting from youngest to oldest - in this manner.	

Section 5.1: Deceased information, 2019 – Part 1

MO05	MO06	MO07	MO08	MO09	MO10	MO11	MO12	MO13
Name of deceased	When did he/she die?	What was the age of "deceased"	Sex<br><br>1=Male<br>2=Female	How was he/she related with the household head?<br><br>See relation code at page 41	Where did s/he die?<br><br>Home 1<br>Hospital 2<br>Elsewhere 3	Did S/he die from an injury (road accident or other)?<br><br>Yes 1<br><br>No 0>>MO12	Please specify the cause of death?<br><br>(See causes of deaths code at page 40)<br>If MO12=other, please specify	Did a medical doctor or nurse examine?<br><br>Yes 1<br><br>No 0

© 2021 Barnwal P et al. *JAMA Network Open.*

1.<br>2.<br>3.<br>4.								

MO14 Note: If MO12=Lung disease, please continue section 5.2

Section 5.2: Deceased information, 2019 – Part 2

MO05	MO14.01	MO14.02	MO14.03	MO14.04	MO14.05	MO14.06	MO14.07	MO14.08	MO14.09
Name of deceased	In the week before dying have a fever?<br>Yes 1<br>No 0	In the week before dying have a headache or dizziness?<br>Yes 1<br>No 0	In the week before dying have a cough?<br>Yes 1<br>No 0	In the week before dying have a sore throat?<br>Yes 1<br>No 0	In the week before dying short of breath or have any difficulty breathing?<br>Yes 1<br>No 0	In the week before dying lose his/her sense of taste or smell?<br>Yes 1<br>No 0	In the week before dying have muscle aches?<br>Yes 1<br>No 0	In the week before dying have a rash?<br>Yes 1<br>No 0	In the week before dying have chills or shake repeatedly with chills?<br>Yes 1<br>No 0
1.<br>2.<br>3.<br>4.									

"Note: I'm sorry hear about your loss. Now I would like to ask you to list all current household members. (Move to next section)"

© 2021 Barnwal P et al. *JAMA Network Open.*

Section 6: Mortality section 2020

Sl	Question	Answer
MO01	Note for enumerator: Respondent didn't mention about some member we listed earlier. Please ask about them - whether anyone of them passed away or migrated in the following section	
MO02	Would you please tell, how many members of your household passed away since January 1st, 2020?	|_________| members "IF 0>>MO02.01 and END OF SECTION
MO02.1	IF MO02=0) Note: I am glad no one died in 2020.	
MO03	"Please tell me the names of your HH member who passed away in 2020.<br><br>Select all that apply."	Other (not in the list): -96>>MO04<br><br>Name 11<br><br>Name 22<br><br>Name 33
MO04	Ask detail about member who died in 2020 - starting from youngest to oldest - in this manner.	

Section 6.1: Deceased information, 2020 – Part 1

MO05	MO06	MO07	MO08	MO09	MO10	MO11	MO12	MO13
Name of deceased	When did he/she die?	What was the age of "deceased"	Sex<br><br>1=Male<br>2=Female	How was he/she related with the household head?<br><br>See relation code at page 41	Where did s/he die?<br><br>Home 1<br>Hospital 2<br>Elsewhere 3	Did S/he die from an injury (road accident or other)?<br><br>Yes 1<br><br>No 0>>MO12	Please specify the cause of death?<br><br>(See causes of deaths code at page 40)<br>If MO12=other, please specify	Did a medical doctor or nurse examine?

© 2021 Barnwal P et al. *JAMA Network Open.*

1.<br>2.<br>3.<br>4.								

MO14 Note: If MO12=Lung disease, please continue section 6.2

Section 6.2: Deceased information, 2020 – Part 2

MO05	MO14.01	MO14.02	MO14.03	MO14.04	MO14.05	MO14.06	MO14.07	MO14.08	MO14.09
Name of deceased	In the week before dying have a fever?<br><br>Yes 1<br>No 0	In the week before dying have a headache or dizziness?<br><br>Yes 1<br>No 0	In the week before dying have a cough?<br><br>Yes 1<br>No 0	In the week before dying have a sore throat?<br><br>Yes 1<br>No 0	In the week before dying short of breath or have any difficulty breathing?<br>Yes 1<br>No 0	In the week before dying lose his/her sense of taste or smell?<br><br>Yes 1<br>No 0	In the week before dying have muscle aches?<br>Yes 1<br>No 0	In the week before dying have a rash?<br>Yes 1<br>No 0	In the week before dying have chills or shake repeatedly with chills?<br><br>Yes 1<br>No 0
1.<br>2.<br>3.<br>4.									

"Note: I'm sorry hear about your loss. Now I would like to ask you to list all current household members. (Move to next section)"

Section 7: Economic impact of covid-19

Note: Thank you for answering the questions. In the following part, I would like to ask a few questions about expenditure and main earning source of the HH

**Sl**	**Question**	**Answer**

© 2021 Barnwal P et al. *JAMA Network Open.*

EC01	Was your household able to buy essential food items over the past 7 days?	Yes >> EC04<br><br>No >> EC02
EC02	Why were you unable to buy these items?<br><br>[READ ALL OPTIONS - SELECT ALL THAT APPLY]	Some items were not available….1<br><br>Some items were more expensive than usual…. 2<br><br>Markets/shops were closed….3<br><br>You did not have enough money (e.g., lack of resources) ….4 >>EC03<br><br>None of the above….5
EC03	In the past 7 days, did you use any of the following to cover your household's basic needs?<br><br>[READ OUT ALL OPTIONS - SELECT ALL THAT APPLY]	Look for ways to earn additional money (e.g., work more hours, do an occasional job, etc.) ….1<br><br>Reduce the number or size of meals for some household members ….2<br><br>Rely on less preferred and less expensive foods….3<br><br>Use cash or bank savings….4<br><br>Sell assets….5<br><br>Borrow food or ask for help from a friend or relative ….6<br><br>Rely on Government or NGO assistance ….7<br><br>Donations…. 8<br><br>None of the above….9
EC04	Who is the usual bread earner in your HH?<br><br>Select one member	Select from the household member list
EC05	"In the past 7 days, have "usual bread earner" worked for remuneration for at least one hour? By ""work for renumeration "" we mean any activities you undertook for remuneration, including daily labor, working for wages or in-kind, or working on your own account or running a business, including an agricultural business. "	Yes 1<br><br>No 0<br><br>Refused -97<br><br>Don't know -99

© 2021 Barnwal P et al. *JAMA Network Open.*

EC06	Type of main occupation of HH member	Select from occupation code
EC06.01	Specify the type of main occupation	Select from type of occupation code
EC06.02	If other, please specify the occupation of	|________________________________|
EC06.03	Wage/ Labor: Other wage labor (specify)	|________________________________|
EC06.04	Salaried worker: Another salaried worker(specify)	|________________________________|
EC06.05	Farming: Other self-employed (specify)	|________________________________|
EC07	Which of the following best describes "usual bread earner" main job?	1- I work for wages or payment (either in cash or in kind) for a company, the government or any other individual<br>2- I do not work for wages, but rather work on my own account or in a business enterprise or farm belonging to me or someone else in my household, for example, as a farmer, trader, shop-keeper, barber, dressmaker, carpenter, or taxi driver
EC08	If EC07=1, Could you tell me what is "usual bread earner" salary/wage from main occupation in an ordinary month, approximately at this time of the year?	|__|__|__|__|__|__| BDT >>EC11<br>Don't know -99 >> EC09
EC09	Could you tell me what is "usual bread earner" wage/salary in an ordinary week or day, approximately at this time of the year?	Yes, I know what was my salary for an ordinary week……2>>EC10<br>Yes, I know my salary for an ordinary day……1>>EC10<br>No……0 >> EC11
EC010	"What is "usual bread earner" wage/salary in an ordinary day/week, approximately at this time of the year?<br>If in the previous section option1 is selected then ask about weekly salary. If option2 selected, ask about per day wage. "	|__|__|__|__|__|__| BDT
EC011	What was "usual bread earner" salary/wage from main occupation in the past month?	|__|__|__|__|__|__| BDT >> EC21<br>Don't know -99 >> EC12
EC012	Do you know what was "usual bread earner" wage/salary in the past day or week?	Yes, I know what was my salary last week…….2>>EC13<br>Yes, I know my salary last day…….1>>EC13

© 2021 Barnwal P et al. *JAMA Network Open.*

		No……. 0 >> EC21
EC013	What was "usual bread earner" wage/salary in the past day/week?<br><br>If in the previous section option1 is selected then ask about weekly salary. If option2 selected, ask about per day wage. "	|__|__|__|__|__|__|__| BDT
EC014	Could you tell me what was "usual bread earner" profit from this farm/enterprise/business in an ordinary month, approximately at this time of the year?	|__|__|__|__|__|__|__| BDT >>EC17<br>Don't know -99 >> EC15
EC015	Could you tell me what was "usual bread earner" profit from this farm/enterprise/business in an ordinary week or day, approximately at this time of the year?	Yes, I know the value of my profit in an ordinary week….2>>EC16<br>Yes, I know the value of my profit in an ordinary day…..1>>EC16<br>No …….0 >> EC17
EC016	What was "usual bread earner" profit from this farm/enterprise/business in an ordinary week or day, approximately at this time of the year?	|__|__|__|__|__|__|__| BDT
EC017	Could you tell me what was "usual bread earner" profit from this farm/enterprise/business last month?	|__|__|__|__|__|__|__| BDT >>EC20<br>Don't know -99 >> EC18
EC018	Could you tell me what was "usual bread earner" profit from this farm/enterprise/business in the last week or day?	Yes, I know the value of my profit in the last week…..2>>EC19<br>Yes, I know the value of my profit in the last day…..1>>EC19<br>No …..0 >> EC20
EC019	What was your profit from this farm/enterprise/business in the last week or day?	|__|__|__|__|__|__|__| BDT
EC020	Do you think "usual bread earner" will be able to pay for all of business expenses (including employees' salaries) next month?	Yes 1<br>No 0
EC021	Do you support Bangladesh government's lockdown decision?	Yes 1<br>No 0<br>Don't know -99

© 2021 Barnwal P et al. *JAMA Network Open.*

EC022	Has any member of your family has lost his/her job/work due to COVID-19 lockdown?	|__|__| People
EC023	Could you estimate how much total household monthly income is lost is your HH due to COVID-19 lockdown?	|___________| taka

© 2021 Barnwal P et al. *JAMA Network Open.*

Phone Survey Oct-Dec 2020

Survey Questionnaire/ Relevant module

Section 1: Survey Tracking Sheet

**Sl**	**Question**	**Answer**
TR1	Date of interview (dd/mm/yy):	[___|___]/[___|___]/[___|___]
TR2	Start time (hh:mm; 24-hour clock):	[___|___] : [___|___]
TR3	Interviewer ID/Name (First Middle Last):	[___|___]________________________________
TR4	Household ID/ Case ID:	[____|____|____]
TR5.1	The HH address from survey was<br>District:<br>Upazila:	 [__|__]<br>[__|__]
TR5.2	Union/Ward:	[__|__]
TR5.3	Village:	[__|__]
TR5.4	Para:	________________
TR5.5	Landmark	________________
TR5.6	Household head	________________

TR 6. Phone response: Please call the household head at given contact numbers.

Instruction for enumerator: Search primarily for household head or respondent of the Census survey. See If any of them are available. First preference for selecting respondent would be female adult member. If the female adult member is busy or not available then we would prefer respondent of the Census survey. If respondent of the Census survey not found then we would prefer household head. If any children pick up the call ask, Is any of adult female member (name of household head or respondent of the Census survey available)? If yes, try to interview one of them.

© 2021 Barnwal P et al. *JAMA Network Open.*

Result of HH Search 1: [__]	Correct household and respondent found 1	
Result of HH Search 2: [__]	No interview: Respondents did not pick up................... 2	>> Try next phone number
Result of HH Search 3: [__]	No interview: Phone was switched off 3	>> Try next phone number
Result of HH Search 4: [__]	No interview: Respondent will not be available during survey period 4	>> Try next phone number
Result of HH Search 5: [__]	No interview: Respondent available at a later time… 5	>> Reschedule
Result of HH Search 6: [__]	No interview: Refusal to talk… 6	>> Try next phone number
Result of HH Search 7: [__]	No interview: Wrong household/ Wrong number.……7	>> TR6.2

Instruction for enumerator: If given contact number is belong to a neighbor. Please ask following.

Note: We surveyed your HH earlier and calling you again this time to ask about one of neighbor.

**Sl**	**Question**	**Answer**
TR6.2	If TR6.1=7, Do you know any of {census's respondent name} ${household head name}? In Jan 2020, they lived in the location above.	Yes……1 >>TR6.3<br>No…… 0 >>Try next phone number
TR6.3	Could you please give us a number for {census's respondent name} ${household head name}?	Yes 1 >>TR6.3.1 No 0
TR6.3.1	Please share the number	[__________________________]
TR6.4	[For Enumerator. Do not read aloud]<br>If neighbor received then, did you collect following information from neighbor - about the household head's location, 2019/2020 deaths related information etc.?	Yes 1 >>Try next phone number<br>No 0

Section 2: Neighbor questions

Note for enumerator: Please ask two neighbors about household head's household location, 2019/2020 deaths related information etc.

© 2021 Barnwal P et al. *JAMA Network Open.*

Sl	Question	Answer
NQ1	I would like to request a few a question about household head's household that we are collecting for research purpose only.	
NQ2	Does the HH still live there?	Yes… ..1<br>No……0 >>NQ2.1
NQ2.1	Could you please tell me, where did the HH moved from here after Jan 2020?	Moved temporarily… .... 1<br>Moved outside of the village permanently.................... 2<br>Moved somewhere else in the village permanent .... 3<br>All HH member died.. 4<br>Other…………………………………………………-96 >>NQ2.1.1
NQ2.1.1	Please specify	
MR1	Are you aware of whether any member that HH has passed away in between Jan, 2019 to Dec, 2019? How many?<br>Write "0" If no one died. If the neighbor doesn't know, write "-99"	|________| members<br>If "0" or "-99" >> MR6

Note: I would like to request you to answer a few questions about the member died in 2019 (Jan - Dec) in that HH.

Instruction for enumerator: Ask details about member who passed away in 2019- starting from youngest to oldest - in this manner

	Question	First deceased	Second deceased	Nth deceased
MR2	Please tell me the name of the … person who died in 2019?	________________________	________________________	________________________
MR3	What was the age of … deceased? "Integer"	|___|___| years	|___|___| years	|___|___| years
MR4	What was the sex of … deceased?	Male………1<br>Female……2	Male………1<br>Female……2	Male………1<br>Female……2

© 2021 Barnwal P et al. *JAMA Network Open.*

MR5	When did … die? "Select date, month, year."	|___|____|_______| years	|___|____|_______| years	|___|____|_______| years

MR6	Are you aware of whether any member that HH has passed away in 2020? How many?<br>Write "0" If no one died. If the neighbor doesn't know, write "-99"	|________| members<br>If "0" or "-99" >> MR6

Note: I would like to request you to answer a few questions about the member died in 2020 in that HH.

Instruction for enumerator: Ask details about member who passed away in 2020- starting from youngest to oldest - in this manner

Sl	Question	First deceased	Second deceased	Nth deceased
MR7	Please tell me the name of the … person who died in 2020?	Select from the dropdown menu	Select from the dropdown menu	Select from the dropdown menu
MR8	What was the age of … deceased? "Integer"	|___|___| years	|___|___| years	|___|___| years
MR9	What was the sex of … deceased?	Male………1<br>Female……2	Male………1<br>Female……2	Male………1<br>Female……2
MR10	When did … die? "Select date, month, year."	|___|____|_______| years	|___|____|_______| years	|___|____|_______| years

MR11	Has anyone died due to Covid19 in that household in 2020?	Yes 1<br>No 2<br>Don't know -99

NOTE: If the correct household has been reached (i.e., if TR6.1=1), then the following questions will appear.

Section 3: Respondent information and Consent

© 2021 Barnwal P et al. *JAMA Network Open.*

Sl	Question	Answer
**RI1**	Who am I talking with?	[________________________]<br>**Select from the drop-down menu. IF RI1=-96>>RI2**
**RI2**	Name of the respondent	[_____________________________]

R14. CONSENT NOTE (for short survey):

Hello, my name is ${enumname} and I am working with Innovations for Poverty Action (IPA), Bangladesh, a research and policy non-profit organization that trying to help with Rural Household facing problems. Previously we conducted a phone call survey in your HH and collected information about household members health condition, morbidity and current pandemic situation. Now, we are inviting you to take part in this phone call survey as we considered you knowledgeable to answer a few questions about your households current deceased member, current pandemic situation, household assets and with which HH you socialize often. The survey will take around 25 minutes.

We hope that this research will help us better understand the mortality trend and impact of current pandemic of the community in order to improve future interventions directed towards public health improvement. Note that all the information collected will be treated as strictly confidential. You have the right to decline to answer any question and to stop the interview at any time. If you complete the whole survey, you will receive a small mobile balance transfer as a gift. We may contact with your household for further data collection. The information collected will help IPA empower the rural people in the region. If you have any questions regarding our project or want to know in more details, kindly contact Innovations of Poverty Action through+88 01712-121221, Md. Alamgir Kabir.

R15. CONSENT NOTE (for long survey):

Hello, my name is ${enumname} and I am working with Innovations for Poverty Action (IPA), Bangladesh, a research and policy non-profit organization that trying to help with Rural Household facing problems. Previously we conducted a phone call survey in your HH and collected information about household members health condition, morbidity and current pandemic situation. Now, we are inviting you to take part in this phone call survey as we considered you knowledgeable to answer a few questions about your households current deceased member, current pandemic situation, household assets and with which HH you socialize often. The survey will take around 40 minutes.

We hope that this research will help us better understand the mortality trend and impact of current pandemic of the community in order to improve future interventions directed towards public health improvement. Note that all the information collected will be treated as strictly confidential. You have the right to decline to answer any question and to stop the interview at any time. If you complete the whole survey, you will receive a mobile balance transfer as a gift. We may contact with your household for further data collection. The information collected will help IPA empower the rural people in the region. If you have any questions regarding our project or want to know in more details, kindly contact Innovations of Poverty Action through+88 01712-121221, Md. Alamgir Kabir.

© 2021 Barnwal P et al. *JAMA Network Open.*

**RI5**	Did you inform about the above statement to a member of the household who gave the consent to the survey (or to a person knowledgeable about household members)?	Yes….1<br>No…. 2 **>>RI8 and End of the survey**
**RI6**	Gender of Respondent	Male 1<br>Female 2
**RI7**	What is the relation of respondent with ${name_1}?<br>Previously we listed ${name_1} as household head. Please tell me your relation with ${name_1}	(See relationship codes page no=8)
**RI8**	You will not complete the whole survey. Please thank the household member for his or her time. Let the member of the household know that you will call another time as per schedule, then complete the final steps if this survey	
**RI9**	Do you remember talking to our team	Yes 1 >>**RI10**<br>No 0
**RI10**	How long ago was it?	[____] MONTHS

Section 4: Household roster update section

Sl	Question	Answer
**RU03**	Firstly, I would like you to list all current household members. Please tell me names of all current HH members.<br>Please note HH member means who<br>- frequently stays in this HH i.e., lives together and shares kitchen together.<br>- contribute financially and primary decision maker the HH or<br>- temporarily living outside of the HH but rely on HH income and regarding decision making (e.g. Student)<br>Any of the above criteria is sufficient to recognized one as a HH member.	**[DO NOT READ OUT OPTIONS LOUDLY**. Record what respondent say<br><br>Other: not in the list -96>>RU04<br>Name 1<br>Name 2<br>Name 3<br>Name 4

© 2021 Barnwal P et al. *JAMA Network Open.*

RU04	How many HH members were not in the list? "Inter number"	|_____| members
	If RU>=1, Collect newly arrived member's information - starting from the oldest to the youngest in this manner following Section 4.1<br><br>Newly arrived people mean who have come to the household after the listing survey or respondent might forget to mentioned his/her name before.	

Section 4.1: New household member demographic Part 1

RU05	RU06	RU07	RU08	RU09	RU10	RU10.1	RUH10.2
Sl number	Name of HH member?	Sex of HH members<br><br>1.Male<br><br>2.Female	Age of the members<br><br>(In year)	Relationship with household head<br><br>(See relationship codes at page 41)	Type of main occupation of the member<br><br>(See occupation code at page 41)	Specify the type of main occupation of the members<br><br>(See occupation code at page 42)	If RU10.1=other, please specify the occupation of members
1.<br><br>2.<br><br>3.<br><br>4.							

Section 4.1: New household member demographic Part 2

RU10.3	RU1 0.4	RU10.5	RU11	RU11.1	RU12	RU13

© 2021 Barnwal P et al. *JAMA Network Open.*

If RU10=Other wage labor (specify)	If RU10= Other salaried worker(specify)	If RU10=Other self-employed (specify)	Where did the new member arrive from recently?<br><br>(See location codes)	If RU11=Other, Please Specify	When did the new member arrive in the household?	Permanent or a temporary member of your household?<br><br>Permanent 1<br><br>Temporary 2
1.<br><br>2.<br><br>3.<br><br>4.						

Section 5: Mortality section 2019

Note for enumerator: If we have completely surveyed this HH during Phone survey May-Jun 2020, please ignore this section and move on to section 6.

**Sl**	**Question**	**Answer**
MO01	**Note**: Now, I would like to ask you a few questions about the changes in Household composition in last year (2019) as well HH member's heath condition	
MO02	"I am sorry if this is painful to recollect but we need to know this for our study. Did any members of your household pass away in 2019?<br><br>Please note that Household member means Who eat together and live together in the house. Whoever visiting this HH for a short period of time/temporarily, won't be considered as HH member.<br><br>Household head means who recognized as the household head by all the members of the household and who normally makes all decisions of household. "	Yes 1<br><br>No 0 >>MO02.1 & move to section
MO02.1	IF MO02=0) Note: I am glad no one died in 2019.	

© 2021 Barnwal P et al. *JAMA Network Open.*

MO03	"How many members of your HH member passed away in between Jan, 2019 to Dec, 2019? ""Inter Number""<br><br>Enumerator: Ask about the household member who passed away in 2019. HH member means who -<br><br>- who consistently lived together and shared the kitchen together.<br><br>- who contributed financially or made HH decisions.<br><br>- who were temporarily outside of the HH but relied on HH income or regarding decision making. For example, Student living outside.<br><br>Any of the above criteria is sufficient to recognized one as a HH member.	
MO04	Note: Ask details about member who passed away in 2019- starting from youngest to oldest - in this manner.	

Section 5.1: Deceased information, 2019 – Part 1

MO05	MO06	MO07	MO08	MO09	MO10	MO11	MO12	MO13
Name of deceased	When did he/she die?	What was the age of "deceased"	Sex<br><br>1=Male<br>2=Female	How was he/she related with the household head?<br><br>See relation code at page 41	Where did s/he die?<br><br>Home 1<br>Hospital 2<br>Elsewhere 3	Did S/he die from an injury (road accident or other)?<br><br>Yes 1<br><br>No 0>>MO12	Please specify the cause of death?<br><br>See cases of death code at page 40<br><br>If MO12=other, please specify	Did a medical doctor or nurse examine?

© 2021 Barnwal P et al. *JAMA Network Open.*

1.<br>2.<br>3.<br>4.								

MO14 Note: If MO12=Lung disease, please continue section 5.2

Section 5.2: Deceased information, 2019 – Part 2

MO05	MO14.01	MO14.02	MO14.03	MO14.04	MO14.05	MO14.06	MO14.07	MO14.08	MO14.09
Name of deceased	In the week before dying have a fever?<br><br>Yes 1<br>No 0	In the week before dying have a headache or dizziness?<br><br>Yes 1<br>No 0	In the week before dying have a cough?<br><br>Yes 1<br>No 0	In the week before dying have a sore throat?<br><br>Yes 1<br>No 0	In the week before dying short of breath or have any difficulty breathing?<br>Yes 1<br>No 0	In the week before dying lose his/her sense of taste or smell?<br><br>Yes 1<br>No 0	In the week before dying have muscle aches?<br>Yes 1<br>No 0	In the week before dying have a rash?<br>Yes 1<br>No 0	In the week before dying have chills or shake repeatedly with chills?<br><br>Yes 1<br>No 0
1.<br>2.<br>3.<br>4.									

"Note: I'm sorry hear about your loss. Now I would like to ask you to list all current household members. (Move to next section)"

© 2021 Barnwal P et al. *JAMA Network Open.*

Section 6: Mortality section 2020

Sl	Question	Answer
MO01	Note for enumerator: Respondent didn't mention about some member we listed earlier. Please ask about them - whether anyone of them passed away or migrated in the following section	
MO02	Would you please tell, how many members of your household passed away since January 1st, 2020?	|_________| members "IF 0>>MO02.01 and END OF SECTION
MO02.1	IF MO02=0) Note: I am glad no one died in 2020.	
MO03	If the household wasn't surveyed in Phone survey May-Jun 2020, please tell me the names of your HH member who passed away in 2020.<br>If the household was completely surveyed in Phone survey May-Jun 2020, Would you please tell, how many members of your household passed away since May 1st, 2020?<br>Select all that apply.	Other (not in the list): -96>>MO04<br>Name 11<br>Name 22<br>Name 33
MO04	Ask detail about member who died in 2020 - starting from youngest to oldest - in this manner.	

Section 6.1: Deceased information, 2020 – Part 1

MO05	MO06	MO07	MO08	MO09	MO10	MO11	MO12	MO13
Name of deceased	When did he/she die?	What was the age of "deceased"	Sex<br>1=Male<br>2=Female	How was he/she related with the household head?<br>See relation code at page 41	Where did s/he die?<br>Home 1<br>Hospital 2<br>Elsewhere 3	Did S/he die from an injury (road accident or other)?<br>Yes 1<br>No 0>>MO12	Please specify the cause of death?<br>See cases of death code at page 40<br>If MO12=other, please specify	Did a medical doctor or nurse examine?

© 2021 Barnwal P et al. *JAMA Network Open.*

1.<br>2.<br>3.<br>4.								

MO14 Note: If MO12=Lung disease, please continue section 6.2

Section 6.2: Deceased information, 2020 – Part 2

MO05	MO14.01	MO14.02	MO14.03	MO14.04	MO14.05	MO14.06	MO14.07	MO14.08	MO14.09
Name of deceased	In the week before dying have a fever?<br><br>Yes 1<br>No 0	In the week before dying have a headache or dizziness?<br><br>Yes 1<br>No 0	In the week before dying have a cough?<br><br>Yes 1<br>No 0	In the week before dying have a sore throat?<br><br>Yes 1<br>No 0	In the week before dying short of breath or have any difficulty breathing?<br>Yes 1<br>No 0	In the week before dying lose his/her sense of taste or smell?<br><br>Yes 1<br>No 0	In the week before dying have muscle aches?<br>Yes 1<br>No 0	In the week before dying have a rash?<br>Yes 1<br>No 0	In the week before dying have chills or shake repeatedly with chills?<br><br>Yes 1<br>No 0
1.<br>2.<br>3.<br>4.									

"Note: I'm sorry hear about your loss. Now I would like to ask you to list all current household members. (Move to next section)"

© 2021 Barnwal P et al. *JAMA Network Open.*

Section 7: Economic impact of covid-19

Note: Thank you for answering the questions. In the following part, I would like to ask a few questions about expenditure and main earning source of the HH

**Sl**	**Question**	**Answer**
EC01	Was your household able to buy essential food items over the past 7 days?	Yes >> EC04<br><br>No >> EC02
EC02	Why were you unable to buy these items?<br><br>[READ ALL OPTIONS - SELECT ALL THAT APPLY]	Some items were not available….1<br><br>Some items were more expensive than usual…. 2<br><br>Markets/shops were closed….3<br><br>You did not have enough money (e.g., lack of resources) ….4 >>EC03<br><br>None of the above….5
EC03	In the past 7 days, did you use any of the following to cover your household's basic needs?<br><br>[READ OUT ALL OPTIONS - SELECT ALL THAT APPLY]	Look for ways to earn additional money (e.g., work more hours, do an occasional job, etc.) ….1<br><br>Reduce the number or size of meals for some household members ….2<br><br>Rely on less preferred and less expensive foods….3<br><br>Use cash or bank savings….4<br><br>Sell assets….5<br><br>Borrow food or ask for help from a friend or relative ….6<br><br>Rely on Government or NGO assistance ….7<br><br>Donations…. 8<br><br>None of the above….9
EC04	Who is the usual bread earner in your HH?<br><br>Select one member	Select from the household member list

© 2021 Barnwal P et al. *JAMA Network Open.*

EC05	"In the past 7 days, have "usual bread earner" worked for remuneration for at least one hour? By ""work for renumeration "" we mean any activities you undertook for remuneration, including daily labor, working for wages or in-kind, or working on your own account or running a business, including an agricultural business. "	Yes 1<br>No 0<br>Refused -97<br>Don't know -99
EC06	Type of main occupation of HH member	Select from occupation code at page 41
EC06.01	Specify the type of main occupation	Select from type of occupation code at page 42
EC06.02	If other, please specify the occupation of	|___________________________________|
EC06.03	Wage/ Labor: Other wage labor (specify)	|___________________________________|
EC06.04	Salaried worker: Another salaried worker(specify)	|___________________________________|
EC06.05	Farming: Other self-employed (specify)	|___________________________________|
EC07	What was "usual bread earner" salary/wage from main occupation in the past month?	|__|__|__|__|__|__|__| BDT >> EC21<br>Don't know -99 >> EC12
EC08	Could you tell me what was "usual bread earner" profit from this farm/enterprise/business last month?	|__|__|__|__|__|__|__| BDT >>EC20<br>Don't know -99 >> EC18
EC09	Do you think "usual bread earner" will be able to pay for all of business expenses (including employees' salaries) next month?	Yes 1<br>No 0
EC010	Do you believe Bangladesh government manage the pandemic well?	Yes 1<br>No 0<br>Don't know -99
EC011	Between April-September, what was the change in total HH income, compared to the previous year?	Income remain same…..1<br>Income has increased……2<br>Income has decreased…….3

© 2021 Barnwal P et al. *JAMA Network Open.*

EC012	During October month, what was the change in total HH income, compared to the same month in previous year?	Income remain same…..1<br>Income has increased……2<br>Income has decreased…….3

Section 8: Asset section

At this part of the survey, I would like request you to answer a few questions regarding you HH assets.

© 2021 Barnwal P et al. *JAMA Network Open.*

Sl	Question	Answer
HA01	Who is the usual bread earner in your HH?	Select one member from the drop-down menu.
HA01.1	In the past week, How many hours per day did main earner ${hh_main_earner} ${hh_main_earner_specify} stay without work ? *"In Hours"*	|__|__| Hours
HA02	How many rooms are there in your house?	|__|__| Rooms
HA03	Does your household have access to electricity/solar?	Yes…1<br>No….0
HA04	How much is the monthly electricity expense of the household? *"Ask the past month expense"*	|_______| Taka
HA05	What type of cookstove is mainly used for cooking?	Electric stove 1<br>Solar cooker 2<br>Liquefied petroleum gas/Cooking gas stove 3<br>Piped natural gas stove 4<br>Biogas stove 5<br>Liquid fuel stove 6<br>Manufactured solid fuel stove 7<br>Traditional solid fuel stove 8<br>Open fire 9<br>No food cooked in the household 10
HA06	What kind of toilet facility do members of your household usually uses?	Flush to piped sewer system 1<br>Flush to septic tank 2<br>Flush to pit latrine 3<br>Flush to somewhere else 4

© 2021 Barnwal P et al. *JAMA Network Open.*

		Ventilated improved pit latrine 5<br>Pit latrine with slab 6<br>Pit latrine without slab/open pit 7<br>Composting toilet 8<br>Bucket toilet 9<br>Hanging toilet/hanging latrine 10<br>No facility/Bush/Field 11
HA07	If HA03=1, How many fans the household owns?	|__|___| Fans
HA08	How many total mobile phones own by household members?	|__|___| phones >> If HA08>0 then HA09
HA09	If HA08>0, How many of these are smart phones?	|__|___| Smartphones
HA10	How many bi-cycles or rickshaws are owned by household?	|__|___| Bi-cylce/Rickshaw
HA11	How many motor-cycles/auto or easy bikes are owned by household?	|__|___| Motor-bike/Easy bike
HA12	How many vehicle such as cars/trucks/vans are owned by household?	|__|___| Vehicles
HA13	If HA03=1, How many TV are owned by the household?	|___|___| TV
HA14	How many computers/Laptops are owned by the household?	|__|__| Computer/Laptop
HA15	If HA03=1, How many refrigerators are owned by the household?	|__|__| Refrigeretor
HA16	What is the type of household's roof top?	No roof 1<br>Grass 2<br>Soil 3<br>Plastic/polytheen 4<br>Plum/bamboo 5<br>Brick 6<br>Stone 7

© 2021 Barnwal P et al. *JAMA Network Open.*

		Metal 8<br>Wood 9<br>Concrete 10<br>Tiles 11<br>Tin 12<br>Others -96
HA16a	If HA16=-96, Please specify	
HA17	What is the type of household's wall?	No wall1<br>Soil 2<br>Grass 3<br>Soil with stone 4<br>Ply wood 5<br>Card board 6<br>Raw wood 7<br>Concrete 8<br>Stone with concrete 9<br>Clean burn brick 10<br>Cement block 11<br>Polished wood sheets 12<br>Tin 13<br>Others -96
HA17a	If HA17=-96, Please specify	
HA18	How many acres of agricultural land does the household own? *"In Acres"*	|___|___| Acres

HA19	Any of your household members own any of the following insurances?	Crop insurance 1<br>Cattle insurance 2<br>Life insurance 3<br>Health insurance 4<br>Driving insurance 5<br>Other -96<br>None -99
HA19a	If HA19=-96, Please specify	

Causes of deaths code	
1	Cancer
2	Lung disease
3	Heart Disease
4	Stroke
5	Pregnancy related
6	Tuberculosis
7	Diabetes
8	Liver disease
-96	Other

Location codes	
1	Barisal
2	Chittagong
3	Dhaka
4	Khulna
5	Mymensingh
6	Rajshahi
7	Rangpur
8	Sylhet
9	From abroad
10	New birth/adoption
11	Correction: forget to mention this person

© 2021 Barnwal P et al. *JAMA Network Open.*

**Relationship codes**	
1	Household head
2	Spouse of household head
3	Daughter of HH head
4	Mother of HH head
5	Daughter-in-law of HH head
6	Sister of HH head
7	Sister-in-law of HH head
8	Aunt of HH
9	Son of HH head
10	Father of HH head
11	Son-in-law of HH head
12	Brother of HH head
13	Brother-in-law of HH head
14	Uncle of HH
15	Cousin of HH head
16	Niece/Nephew of HH head
17	Grandchildren of HH head
18	Grandparents HH head
19	In-laws of HH
-96	Other

**Education codes**	
0	None
1	Class I
2	Class II
3	Class III
4	Class IV
5	Class V
6	Class VI
7	Class VII
8	Class VIII
9	Class IX
10	SSC/ Dakhil
11	HSC 1st year/ Alim 1st year
12	HSC 2nd year/ Alim 2nd year
13	BA/BSC 1st year/ Fazil 1st year
14	BA/BSC 2nd year/ Fazil 2nd year
15	BA/BSC 3rd year/ Fazil 3rd year
16	BA/BSC 4th year/ Fazil 4th year
17	MA/MSC and above/ Kamil
66	Preschool class (general)/ Preschool (mosque based)
-96	Others (specify)

**Occupation type codes**	
1	Wage/labor
2	Salaried worker
3	Self-employment
4	Trader
5	Production
6	Livestock Poultry related work/occupation
7	Farming
8	Non-earning occupation

**Marital status codes**	
1	Married, living in own house
2	Married, living in in-laws house
3	Married, living in his/her parents house
4	Widowed/widower, living in her/his in-laws house
5	widowed/widower, living in her/his parents house
6	Widowed/widower, living in own house
7	Separated/Divorced
8	Unmarried

© 2021 Barnwal P et al. *JAMA Network Open.*

Occupation codes	
1	Wage/ Labor : Agricultural day labor
2	Wage/ Labor : Earth work (govt program)
3	Wage/ Labor : Earth work (other)
4	Wage/ Labor : Sweeper
5	Wage/ Labor : Scavenger
6	Wage/ Labor : Tea garden worker
7	Wage/ Labor : Construction labor
8	Wage/ Labor : Factory worker
9	Wage/ Labor : Garment worker
10	Wage/ Labor : Fish processing worker
11	Wage/ Labor : Transport worker (bus/truck helper)
12	Wage/ Labor : Hotel/restaurant worker
13	Wage/ Labor : Apprentice
14	Wage/ Labor : Other wage labor (specify)
15	Salaried worker : Government/ parastatal
16	Salaried worker : Service (private sector)
17	Salaried worker : NGO worker
18	Salaried worker : Domestic worker
19	Salaried worker : Teacher (GoB-Primary school)
20	Salaried worker : Teacher (Non GoB Primary school)
21	Salaried worker : Teacher (GoB High school)
22	Salaried worker : Teacher (Non-GoB High school)
23	Salaried worker : Teacher (College/University)
24	Salaried worker : Other salaried worker(specify)
25	Self-employment : Rickshaw/van pulling
26	Self-employment : Driver of motor vehicle
27	Self-employment : Tailor/seamstress
28	Self-employment : Blacksmith

© 2021 Barnwal P et al. *JAMA Network Open.*

29	Self-employment : Potter
30	Self-employment : Cobbler
31	Self-employment : Hair cutter
32	Self-employment : Clothes washer
33	Self-employment : Porter
34	Self-employment : Goldsmith/Silversmith
35	Self-employment : Repairman (appliances)
36	Self-employment : Mechanic (vehicles)
37	Self-employment : Plumber
38	Self-employment : Electrician
39	Self-employment : Clothes washer
40	Self-employment : Carpenter
41	Self-employment : Mason
42	Self-employment : Doctor
43	Self-employment : Rural physician
44	Self-employment : Midwife
45	Self-employment : Herbal doctor/Kabiraj
46	Self-employment : Electrician :Engineer
47	Self-employment : Lawyer/deed writer/Moktar
48	Self-employment : Religious leader (Imam/Muazzem/Khadem/Purohit)
49	Self-employment : Lodging master
50	Self-employment : Private tutor/house tutor
51	Self-employment : Beggar
52	Trader :Small trader (roadside stand or stall)
53	Trader :Medium trader (shop or small store)
54	Trader :Large trader (large shop or whole sale)
55	Trader :Fish Trader
56	Trader :Wild fish seed collector
57	Trader :Contractor
58	Production :Food Processing
59	Production :Small industry

© 2021 Barnwal P et al. *JAMA Network Open.*

60	Production :Handicrafts

61	Livestock Poultry related work/occupation :Milk collector
62	Livestock Poultry related work/occupation :Livestock Vet medicine seller
63	Livestock Poultry related work/occupation :Livestock Feed supplier
64	Livestock Poultry related work/occupation :Commercially feed producer
65	Livestock Poultry related work/occupation :Animal Breeder
66	Livestock Poultry related work/occupation :Veterinary/paravet doctor
67	Farming :Working own farm (crop)
68	Farming :Share cropper/tenant
69	Farming :Homestead farming
70	Farming :Fishers (using non owned/not Leased water body)
71	Farming :Fish farmer/producer
72	Farming :Nursery owner
73	Farming :Fish hatchery owner
74	Farming :Raising poultry
75	Farming :Raising livestock
76	Farming :Dairy production/dairy farming
77	Farming :Other self-employed (specify)
78	Non-earning occupation :Own household domestic duties
79	Non-earning occupation :Not working but seeking and is available for work
80	Non-earning occupation :Unable to work due to ill- health/disability
81	Non-earning occupation :Not employed and not seeking work such as landlords, rentiers, pensioners, remittances
82	Non-earning occupation :Senior citizen/ child below age 5
83	Non-earning occupation :Student/trainings
-96	Others (specify)

© 2021 Barnwal P et al. *JAMA Network Open.*
